# Supplementary material for: Re-examining the relationship between invasive lionfish and native grouper in the Caribbean
Source: PeerJ. 2014 Apr 15;2:e348. doi: 10.7717/peerj.348 (PMC3994649; doi:10.7717/peerj.348)
Supplement: Table S1 — Location names, coordinates, and site characteristics of surveyed sites. S&G, spur-and-groove. [file peerj-02-348-s001.docx]

| **Country** | **Sub Region** | **Latitude** | **Longitude** | **Site Name** | **Site Code** | **Depth (m)** | **Survey Year** | **Windward/ Leeward** | **Protection (y/n)** | **Habitat type** |
| --- | --- | --- | --- | --- | --- | --- | --- | --- | --- | --- |
| Bahamas | Abaco | 25.99661 | -77.4009 | Rocky Point Slope | RP | 11.00 | 2011 | Leeward | n | Slope |
| Bahamas | Abaco | 26.04617 | -77.4773 | Sandy Point | SP | 3.00 | 2011 | Leeward | n | Patch |
| Bahamas | Abaco | 26.3239 | -76.9916 | Little Harbor | LH | 11.00 | 2011 | Windward | n | S&G |
| Bahamas | Abaco | 26.39783 | -76.9885 | Pelican Cay | PC | 8.00 | 2011 | Leeward | y | Slope |
| Bahamas | Abaco | 26.62122 | -77.0055 | Man o War | MW | 11.00 | 2011 | Windward | n | S&G |
| Bahamas | Abaco | 26.63717 | -77.0385 | Fowls Cay | FC | 8.00 | 2011 | Leeward | y | Slope |
| Bahamas | Abaco | 26.70967 | -77.1541 | Guana Cay | GC | 11.00 | 2011 | Windward | n | S&G |
| Bahamas | Eleuthera | 24.8174 | -76.2442 | 108 | 108 | 2.44 | 2009 | Leeward | n | Patch |
| Bahamas | Eleuthera | 24.81817 | -76.2495 | 106 | 106 | 3.05 | 2009 | Leeward | n | Patch |
| Bahamas | Eleuthera | 24.8194 | -76.261 | 94 | 94 | 3.05 | 2009 | Leeward | n | Patch |
| Bahamas | Eleuthera | 24.82102 | -76.2573 | 112 | 112 | 3.05 | 2009 | Leeward | n | Patch |
| Bahamas | Eleuthera | 24.82422 | -76.2533 | 102 | 102 | 2.64 | 2009 | Leeward | n | Patch |
| Bahamas | Eleuthera | 24.82643 | -76.2483 | 101 | 101 | 3.05 | 2009 | Leeward | n | Patch |
| Bahamas | Eleuthera | 24.82758 | -76.2663 | 104 | 104 | 3.05 | 2009 | Leeward | n | Patch |
| Bahamas | Eleuthera | 24.82792 | -76.2438 | 100 | 100 | 3.05 | 2009 | Leeward | n | Patch |
| Bahamas | Eleuthera | 24.83282 | -76.2727 | 71 | 71 | 3.05 | 2009 | Leeward | n | Patch |
| Bahamas | Eleuthera | 24.83398 | -76.2429 | 84 | 84 | 3.05 | 2009 | Leeward | n | Patch |
| Bahamas | Eleuthera | 24.8367 | -76.2608 | 93 | 93 | 3.05 | 2009 | Leeward | n | Patch |
| Bahamas | Eleuthera | 24.84107 | -76.2445 | 80 | 80 | 3.05 | 2009 | Leeward | n | Patch |
| Bahamas | Eleuthera | 24.84423 | -76.2473 | 79 | 79 | 2.95 | 2009 | Leeward | n | Patch |
| Bahamas | Eleuthera | 24.84454 | -76.2604 | 55 | 55 | 3.05 | 2009 | Leeward | n | Patch |
| Bahamas | Eleuthera | 24.84635 | -76.2542 | 90 | 90 | 2.95 | 2009 | Leeward | n | Patch |
| Bahamas | Eleuthera | 24.84838 | -76.2422 | 89 | 89 | 3.05 | 2009 | Leeward | n | Patch |
| Bahamas | Eleuthera | 24.84968 | -76.2511 | 91 | 91 | 2.74 | 2009 | Leeward | n | Patch |
| Bahamas | Eleuthera | 24.85208 | -76.2583 | 76 | 76 | 3.05 | 2009 | Leeward | n | Patch |
| Bahamas | Eleuthera | 24.85253 | -76.2551 | 75 | 75 | 3.05 | 2009 | Leeward | n | Patch |
| Bahamas | Eleuthera | 24.85275 | -76.2471 | 78 | 78 | 3.35 | 2009 | Leeward | n | Patch |
| Bahamas | Eleuthera | 24.85302 | -76.2492 | 77 | 77 | 3.25 | 2009 | Leeward | n | Patch |
| Bahamas | Eleuthera | 24.85712 | -76.2574 | 74 | 74 | 3.15 | 2009 | Leeward | n | Patch |
| Bahamas | Eleuthera | 24.85883 | -76.2543 | 73 | 73 | 2.74 | 2009 | Leeward | n | Patch |
| Bahamas | Eleuthera | 24.86077 | -76.2594 | 72 | 72 | 3.35 | 2009 | Leeward | n | Patch |
| Bahamas | Eleuthera | 24.95058 | -76.2871 | 70 | 70 | 3.05 | 2009 | Leeward | n | Patch |
| Bahamas | New Providence | 24.91062 | -77.5263 | RSP Group | RS | 15.40 | 2010 | Leeward | n | S&G |
| Bahamas | New Providence | 24.97067 | -77.5342 | Mike's Reef | MK | 13.00 | 2010 | Leeward | n | S&G |
| Bahamas | New Providence | 24.97443 | -77.5352 | Pumpkin Patch | PK | 11.50 | 2010 | Leeward | n | S&G |
| Bahamas | New Providence | 25.0013 | -77.5416 | Power Plant | PW | 7.00 | 2010 | Leeward | n | S&G |
| Bahamas | New Providence | 25.0045 | -77.5538 | DC3 Wall | DW | 13.75 | 2010 | Leeward | n | S&G |
| Bahamas | New Providence | 25.00617 | -77.552 | David Tucker | DT | 14.50 | 2010 | Leeward | n | S&G |
| Bahamas | New Providence | 25.0085 | -77.5572 | Bond Wrecks | BW | 11.50 | 2010 | Leeward | n | S&G |
| Bahamas | New Providence | 25.01535 | -77.5698 | Willaurie | WI | 15.00 | 2010 | Leeward | n | S&G |
| Belize | Meso American | 16.1123 | -88.2559 | Nicholas | NI | 13.00 | 2012 | Windward | y | S&G |
| Belize | Meso American | 16.11247 | -88.2711 | Southwest | ST | 13.00 | 2012 | Windward | n | S&G |
| Belize | Meso American | 16.28501 | -88.1503 | Ranguana | RA | 13.00 | 2012 | Windward | n | S&G |
| Belize | Meso American | 16.3731 | -88.0891 | Pampion | PO | 13.00 | 2012 | Windward | n | S&G |
| Belize | Meso American | 16.72875 | -87.8287 | South Middle Cay | SM | 13.00 | 2012 | Windward | y | S&G |
| Belize | Meso American | 16.73703 | -87.8054 | Middle Cay | MC | 13.00 | 2012 | Windward | y | S&G |
| Belize | Meso American | 16.81346 | -88.0776 | South Water | SW | 13.00 | 2012 | Windward | y | S&G |
| Belize | Meso American | 16.91911 | -88.0476 | Tobacco | TO | 13.00 | 2012 | Windward | n | S&G |
| Belize | Meso American | 17.1966 | -88.0512 | Alligator | AL | 13.00 | 2012 | Windward | n | S&G |
| Belize | Meso American | 17.2056 | -87.5468 | Half Moon | HM | 13.00 | 2012 | Leeward | y | S&G |
| Belize | Meso American | 17.26147 | -87.8197 | Calabash | CA | 13.00 | 2012 | Windward | n | S&G |
| Belize | Meso American | 17.49592 | -88.0426 | Gallows | GA | 13.00 | 2012 | Windward | n | S&G |
| Belize | Meso American | 17.86343 | -87.9724 | Hol Chan | HC | 13.00 | 2012 | Windward | y | S&G |
| Belize | Meso American | 17.91056 | -87.9508 | Tackle Box | TB | 13.00 | 2012 | Windward | n | S&G |
| Belize | Meso American | 17.98782 | -87.9038 | Mexico Rocks | MR | 13.00 | 2012 | Windward | n | S&G |
| Belize | Meso American | 18.16282 | -87.8222 | Bacalar Chico | BC | 13.00 | 2012 | Windward | n | S&G |
| Cuba | Bay of Pigs | 22.07914 | -81.076 | Ebano | EB | 11.00 | 2010 | Windward | n | Slope |
| Cuba | Bay of Pigs | 22.11003 | -81.1163 | Punta Perdiz | PZ | 9.00 | 2010 | Leeward | n | Slope |
| Cuba | Bay of Pigs | 22.16627 | -81.1383 | Cueva Peces | CP | 9.00 | 2010 | Leeward | n | Slope |
| Cuba | Jardines de la Reina | 20.76177 | -78.8522 | Five Sea | CF | 11.00 | 2011 | Leeward | y | Slope |
| Cuba | Jardines de la Reina | 20.78697 | -78.9432 | Anclita | AN | 11.00 | 2011 | Leeward | y | Slope |
| Cuba | Jardines de la Reina | 20.82586 | -78.9803 | Pipin | PP | 16.00 | 2011 | Leeward | y | S&G |
| Cuba | Jardines de la Reina | 20.84411 | -79.0217 | El Peruano | EP | 11.00 | 2011 | Leeward | y | Slope |
| Cuba | North Coast | 23.14654 | -81.6666 | Bacunayaua | BA | 10.00 | 2012 | Windward | n | Slope |
| Mexico | Akumal | 20.42689 | -87.286 | Xaak | XA | 15.00 | 2012 | Windward | n | S&G |
| Mexico | Banco Chinchorro | 18.41008 | -87.4169 | Banco Chinchorro South | BCS | 15.00 | 2012 | Leeward | y | S&G |
| Mexico | Banco Chinchorro | 18.57457 | -87.4198 | Banco Chinchorro Central | BCC | 15.00 | 2012 | Leeward | y | S&G |
| Mexico | Banco Chinchorro | 18.74867 | -87.3476 | Banco Chinchorro North | BCN | 15.00 | 2012 | Leeward | y | S&G |
| Mexico | Cancun | 21.02544 | -86.7713 | Gardener of Hope | GH | 12.00 | 2012 | Windward | n | S&G |
| Mexico | Cozumel | 20.31961 | -87.0266 | Columbia Reef | CR | 15.00 | 2012 | Leeward | y | S&G |
| Mexico | Cozumel | 20.47188 | -86.9815 | Paraiso Bajo | PB | 10.00 | 2012 | Leeward | y | S&G |
