# Supplementary material for: Re-examining the relationship between invasive lionfish and native grouper in the Caribbean
Source: PeerJ. 2014 Apr 15;2:e348. doi: 10.7717/peerj.348 (PMC3994649; doi:10.7717/peerj.348)
Supplement: Table S2 — Lionfish abundance (ind. 100 m−2) on grouper biomass (g 100 m−2), predators, and other co-factors. [file peerj-02-348-s002.docx]

| Coefficients | Estimate | Std. Error | z value | Pr(>\|z\|) |  |
| --- | --- | --- | --- | --- | --- |
| (Intercept) | 3.19012 | 1.18970 | 2.68 | 0.0073 ** |  |
| Habitat (Spur & Groove) | -3.34259 | 1.44490 | -2.31 | 0.0207 * |  |
| Habitat (Slope) | -2.75324 | 1.33400 | -2.06 | 0.0390 * |  |
| Windward | -1.27082 | 0.63682 | -2.00 | 0.0460 * |  |
| Protection (yes) | -1.32074 | 0.48898 | -2.70 | 0.0069 ** |  |
| Depth | 0.25256 | 0.30394 | 0.83 | 0.4060 |  |
| Time since invasion | 0.32035 | 0.34958 | 0.92 | 0.3595 |  |
| Reef complexity | -0.00503 | 0.07948 | -0.06 | 0.9496 |  |
| Humans/Reef | 0.09210 | 0.08807 | 1.05 | 0.2957 |  |
| Predator biomass | -0.08192 | 0.09066 | -0.90 | 0.3662 |  |
| log(Grouper biomass) | -0.00709 | 0.04494 | -0.16 | 0.8747 |  |
| Significance codes: 0 ‘***’ 0.001 ‘**’ 0.01 ‘*’ 0.05 ‘.’ 0.1 ‘ ’ 1 | | | | | |
|  | | | | | |
| Number of observations: total=363, Region=11, Region: Site.Code=71 | | | | | |
|  | | | | | |
| Random effect variance(s): | | | | | |
| Group=Region | | | | | |
| Variance StdDev | | | | | |
| (Intercept) 1.179 1.086 | | | | | |
|  | | | | | |
| Group=Region/Site.Code | | | | | |
| Variance StdDev | | | | | |
| (Intercept) 0.3861 0.6214 | | | | | |
|  | | | | | |
| Negative binomial dispersion parameter: 1.568 (std. err.: 0.16423) | | | | | |
|  | | | | | |
| Zero-inflation: 0.0083623 (std. err.: 0.0096232 ) | | | | | |
|  | | | | | |
| Log-likelihood: -618.889 AIC: 1267.8 | | | | | |
