# Supplementary figures and images for: Re-examining the relationship between invasive lionfish and native grouper in the Caribbean
Source: PeerJ. 2014 Apr 15;2:e348. doi: 10.7717/peerj.348 (PMC3994649; doi:10.7717/peerj.348)

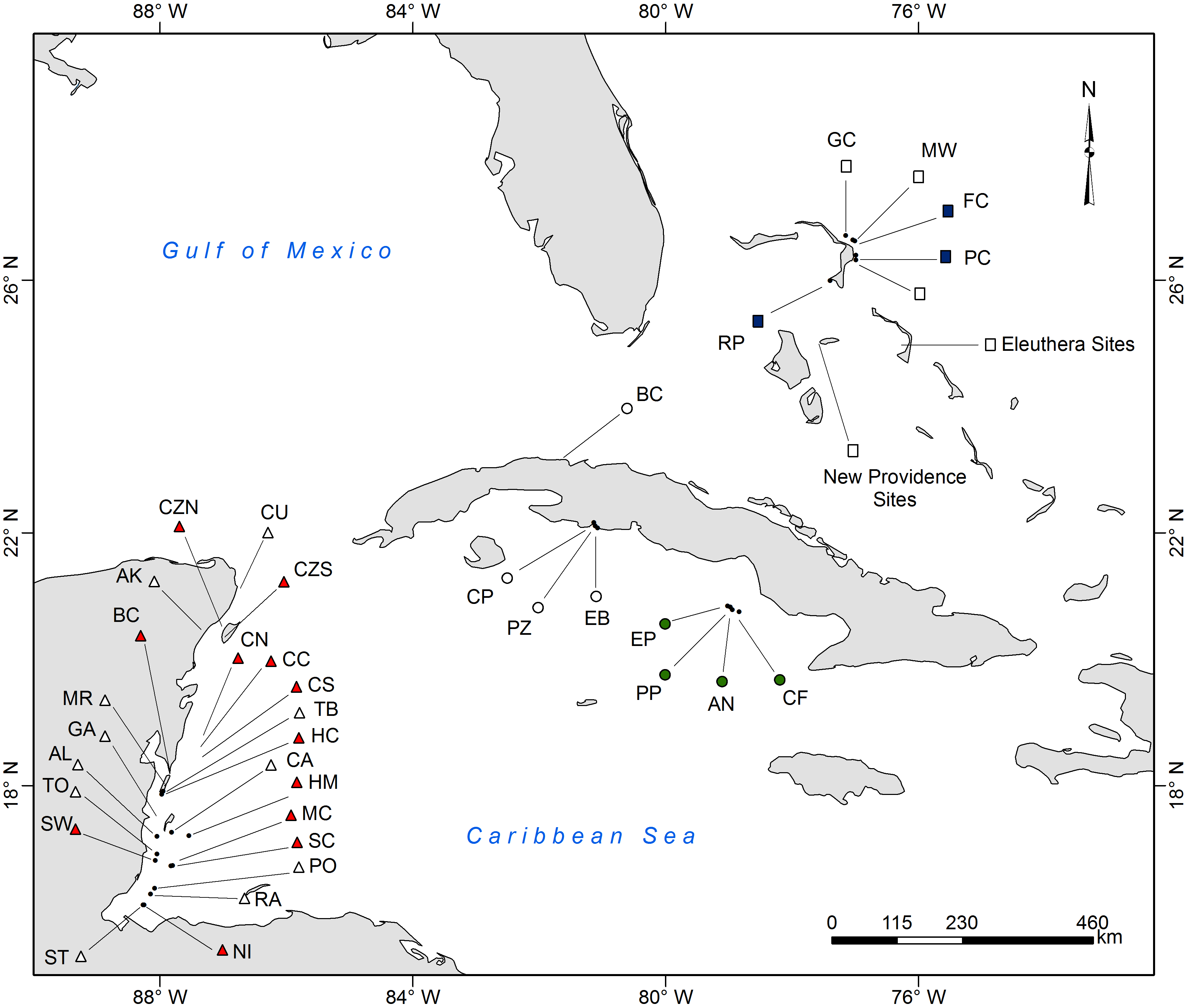

Supplement: Figure S1 — For site abbreviations, surveys dates and coordinates refer to Table S1. [file peerj-02-348-s003.png]

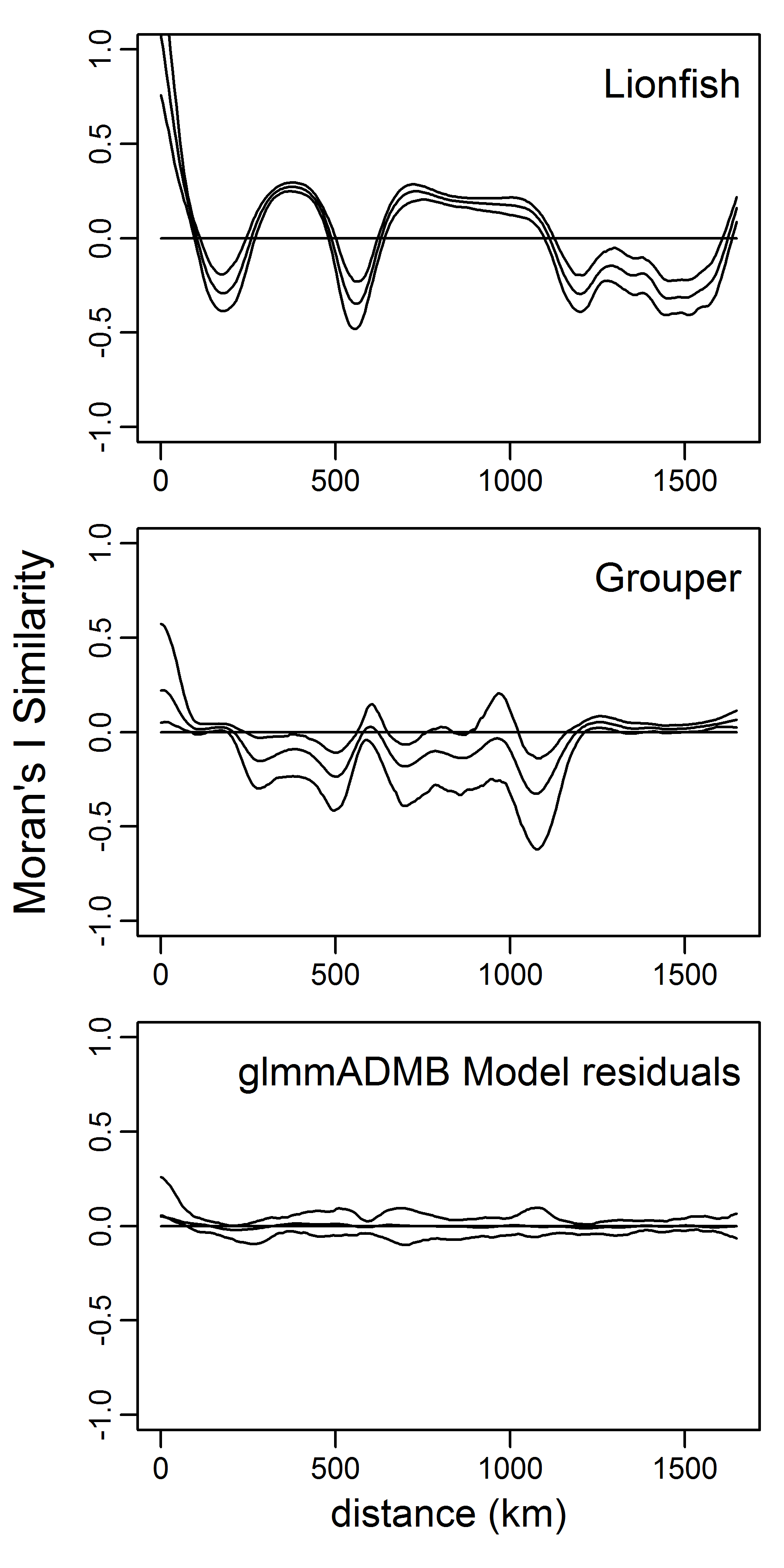

Supplement: Figure S2 — Note the strong spatial autocorrelation of the raw data (i.e., swirling lines around zero) and how the hierarchical structure of the random effects (sites nested in regions) of the full glmmADMB model eliminated this correlation in the model residuals. A Mantel test of the model Pearson residuals (r = 0.073) corroborates the lack of spatial correlation of the residuals. Lines are the mean ±95% confidence interval. [file peerj-02-348-s004.png]

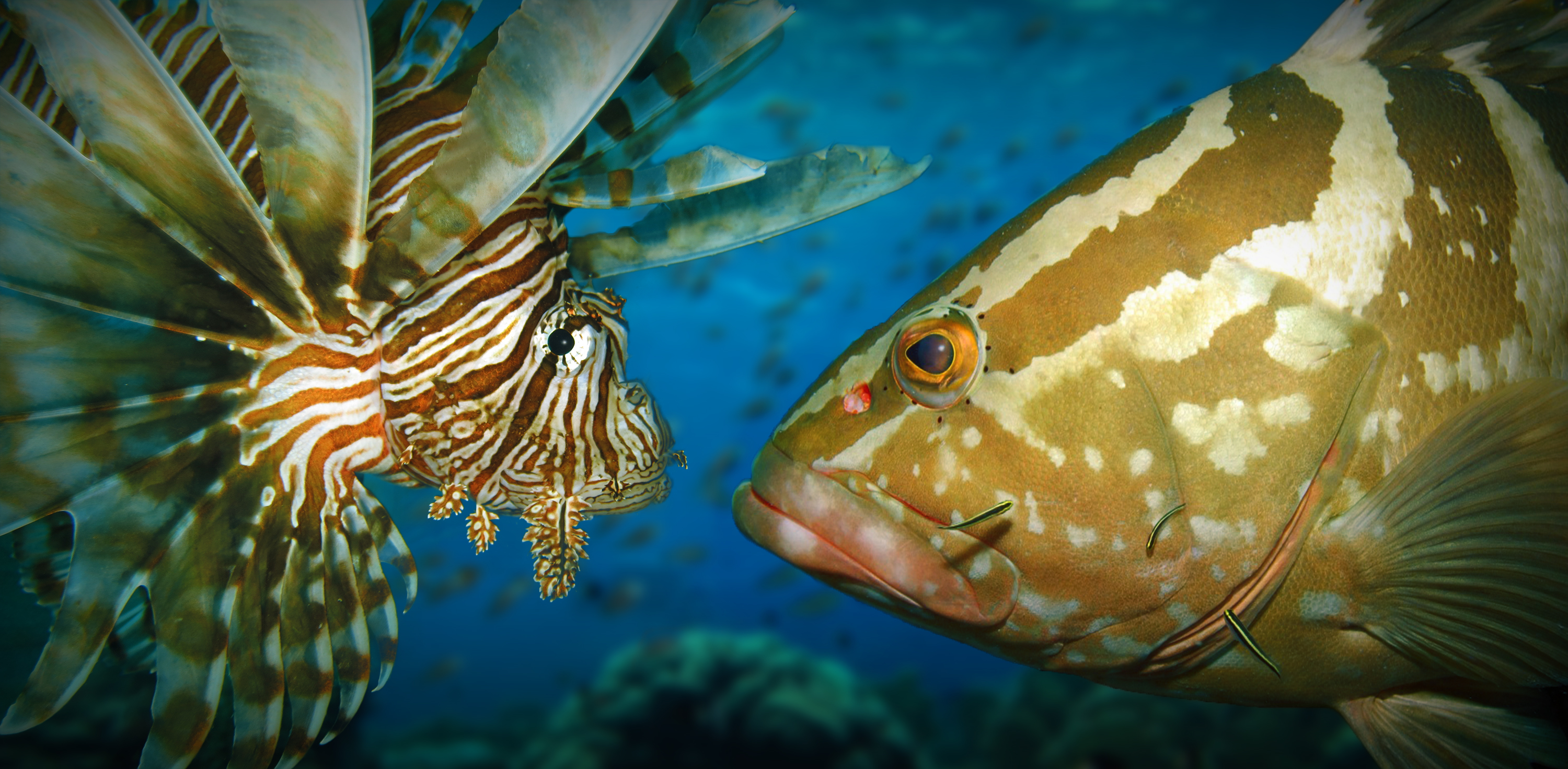

Supplement: Supplemental Information 6 [file peerj-02-348-s006.jpg]
