# Supplementary material for: Re-examining the relationship between invasive lionfish and native grouper in the Caribbean
Source: PeerJ. 2014 Apr 15;2:e348. doi: 10.7717/peerj.348 (PMC3994649; doi:10.7717/peerj.348)
Supplement: Appendix S1 [file peerj-02-348-s005.docx]

**Appendix**

### R Code for Grouper and Lionfish paper

### Written by Abel Valdivia, UNC Chapel Hill###

### Last date edited 01/12/2014###

#Set Working directory

setwd("C:/.../Lionfish and groupers/Data Analysis")

#Load Data

fish=read.csv("./LFdata.csv")

#Check data

str(fish)

#Calculate Large Grouper Biomass and Density with same species that Mumby et al 2011 used

#Include the rest of large predators except groupers as a covariate in the model

#Include it in the data frame

fish <- within(fish, {Grouper.Biom = Black.Biom + Nassau.Biom + Tiger.Biom + Yellowfin.Biom})

fish <- within(fish, {Grouper.Abund=Black.Abund+Nassau.Abund+Tiger.Abund+Yellowfin.Abund})

fish <- within(fish, {Predators = LP.Biom-Grouper.Biom})

#Calculate the log of the survey area to add it as an offset in the model

fish <- within(fish, {LogArea = log(fish$Area4LF)})

#Attach fish data to make coding easier. This might problematic with some code!

attach(fish)

#First explore LF data and check LF count distribution

library (MASS)

#add small value to Y to account for log in the link

dlnorm1<-glm(LF.Count+1e-9~1, family=gaussian, offset=LogArea)

dlnorm2<-glm(LF.Count+1e-9~1, family=gaussian (link="log"), start= 1, offset=LogArea)

dquasi<-glm(LF.Count+1e-9~1, family=quasi, offset=LogArea)

dpois1<- glm(LF.Count+1e-9~1, family=poisson, offset=LogArea)

dqpois1<-glm(LF.Count+1e-9~1, family=quasipoisson, offset=LogArea)

dnbinom<-glm.nb(LF.Count+1e-9~1) #this one has Lower AIC

#Run AIC and BIC to compare among models

AIC(dlnorm1,dlnorm2, dquasi,dpois1,dqpois1,dnbinom)

df AIC

dlnorm1 2 2679.110

dlnorm2 2 2735.708

dquasi 1 NA

dpois1 1 8973.988

dqpois1 1 NA

dnbinom 2 1775.966

BIC(dlnorm1,dlnorm2, dquasi,dpois1,dqpois1,dnbinom)

df BIC

dlnorm1 2 2686.899

dlnorm2 2 2743.497

dquasi 1 NA

dpois1 1 8977.882

dqpois1 1 NA

dnbinom 2 1783.754

#Run an analysis of deviance

>anova(dlnorm1,dlnorm2, dquasi,dpois1,dqpois1,dnbinom)

Analysis of Deviance Table

Model 1: LF.Count + 1e-09 ~ 1

Model 2: LF.Count + 1e-09 ~ 1

Model 3: LF.Count + 1e-09 ~ 1

Model 4: LF.Count + 1e-09 ~ 1

Model 5: LF.Count + 1e-09 ~ 1

Model 6: LF.Count + 1e-09 ~ 1

Resid. Df Resid. Dev Df Deviance

1 362 33724

2 362 39415 0 -5690.3

3 362 33724 0 5690.3

4 362 8263 0 25460.8

5 362 8263 0 0.0

6 362 346 0 7917.0

#Build figure with LF Counts distribution

par(fig=c(0,1,0,0.35))

boxplot(LF.Count, horizontal=T, bty="n", xlab="lionfish counts", ylim=c(0,55), cex.lab=1.2)

par(fig=c(0,1,0.15,1), new=T)

x <- sort(LF.Count)

hist(LF.Count, freq=F, main="", col="darkgray", ylim=c(0,0.7),breaks=seq(0,55,1), xlab="");box()

legend("topright", c("log-normal distribution", "exponential distribution","poisson distribution","nbinom distribution", "density counts"),

lty=c(1,1,3,1,2), lwd=c(1.5,1,3,2,1), col=c(2, "grey30","darkgreen","black", 4), bty="n")

#distributions

lines(density(LF.Count), lty=2, col=4)

#negative binomial

curve(dnbinom(round(x), size=0.5, mu=1), lty=1,lwd=2, col="black", add=T)

#lognormal

curve(dlnorm(x, meanlog=0, sdlog=1), lwd=1.5, add=T, col=2)

#exponential

curve(dexp(x), add=T, lty=1, col="grey30", lwd=1.5)

#poisson

lines(dpois(x,mean(LF.Count)), lty=3, lwd=3, col="darkgreen")

rug(LF.Count, ticksize = 0.02)


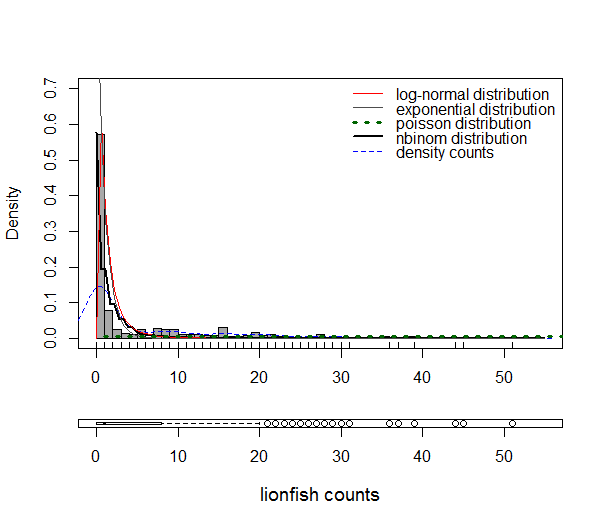


Figure Distribution of lionfish counts

#Try models with Poisson and negative binomial distribution

#Possible negative binomial will be better since ~47% of the count data are zeroes

#Negative binomial accounts for zero inflation, needs a model that accounts for over dispersion

#Run VIF to detect correlation between numerical factors

#Load package (car)

library(car)

#First, run a logistic model with all variable with a default Gaussian distribution

LFvsGrper.Biom.glm= glm(LF.Count ~ Grouper.Biom + Predators

+ Time + Depth + Protection + Habitat + WindvsLee + Rugosity.t. + Hum.Reef,

data=fish, offset = LogArea)

summary(LFvsGrper.Biom.glm)

Call:

glm(formula = LF.Count ~ Grouper.Biom + Predators + Time + Depth +

Protection + Habitat + WindvsLee + Rugosity.t. + Hum.Reef,

data = fish, offset = LogArea)

Deviance Residuals:

Min 1Q Median 3Q Max

-12.41 -1.40 -0.69 0.50 33.25

Coefficients:

Estimate Std. Error t value Pr(>|t|)

(Intercept) 1.170e+01 2.044e+00 5.723 2.24e-08 ***

Grouper.Biom 1.155e-02 2.128e-02 0.543 0.5875

Predators -1.382e-03 2.402e-03 -0.575 0.5654

Time 1.926e-01 3.600e-01 0.535 0.5929

Depth 1.779e-01 2.163e-01 0.822 0.4115

Protectiony -4.605e-01 8.325e-01 -0.553 0.5805

HabitatS&G -1.938e+01 2.494e+00 -7.773 8.53e-14 ***

HabitatSlope -1.843e+01 1.793e+00 -10.282 < 2e-16 ***

WindvsLeeWindward -8.154e-01 8.314e-01 -0.981 0.3274

Rugosity.t. -3.074e-02 3.517e-01 -0.087 0.9304

Hum.Reef 2.159e-04 1.259e-04 1.715 0.0872 .

---

Signif. codes: 0 ‘***’ 0.001 ‘**’ 0.01 ‘*’ 0.05 ‘.’ 0.1 ‘ ’ 1

(Dispersion parameter for gaussian family taken to be 29.06986)

Null deviance: 33724 on 362 degrees of freedom

Residual deviance: 10233 on 352 degrees of freedom

AIC: 2266.2

Number of Fisher Scoring iterations: 2

#AIC=2266.2 Try a poission distribution with log link

#Run a logistic model with all variables with a poisson distribution

LFvsGrper.Biom.glmp= glm(LF.Count ~ Grouper.Biom +Predators

+ Time + Depth + Protection + Habitat + WindvsLee + Rugosity.t. + Hum.Reef,

data=fish, offset = LogArea, family=poisson)

summary(LFvsGrper.Biom.glmp)

Call:

glm(formula = LF.Count ~ Grouper.Biom + Predators + Time + Depth +

Protection + Habitat + WindvsLee + Rugosity.t. + Hum.Reef,

family = poisson, data = fish, offset = LogArea)

Deviance Residuals:

Min 1Q Median 3Q Max

-5.4926 -1.3014 -1.0424 0.6255 8.1257

Coefficients:

Estimate Std. Error z value Pr(>|z|)

(Intercept) -2.921e+00 3.026e-01 -9.654 < 2e-16 ***

Grouper.Biom 1.274e-02 2.063e-03 6.177 6.52e-10 ***

Predators -9.264e-04 4.211e-04 -2.200 0.027817 *

Time 1.049e-01 5.995e-02 1.750 0.080094 .

Depth 8.309e-02 3.366e-02 2.468 0.013573 *

Protectiony -6.823e-01 1.655e-01 -4.123 3.75e-05 ***

HabitatS&G -4.454e+00 3.973e-01 -11.210 < 2e-16 ***

HabitatSlope -4.063e+00 2.708e-01 -15.004 < 2e-16 ***

WindvsLeeWindward -8.497e-01 1.604e-01 -5.298 1.17e-07 ***

Rugosity.t. 6.745e-02 4.347e-02 1.552 0.120739

Hum.Reef 4.169e-05 1.130e-05 3.690 0.000224 ***

---

Signif. codes: 0 ‘***’ 0.001 ‘**’ 0.01 ‘*’ 0.05 ‘.’ 0.1 ‘ ’ 1

(Dispersion parameter for poisson family taken to be 1)

Null deviance: 8263.5 on 362 degrees of freedom

Residual deviance: 1518.5 on 352 degrees of freedom

AIC: 2249

Number of Fisher Scoring iterations: 7

#AIC=2249 lower AIC!

#Run vif to see the variance of each factor and potential correlations problems

vif(LFvsGrper.Biom.glm)

GVIF Df GVIF^(1/(2*Df))

Grouper.Biom 1.352594 1 1.163011

Predators 1.382687 1 1.175877

Time 1.531990 1 1.237736

Depth 12.101803 1 3.478765

Protection 1.808929 1 1.344964

Habitat 17.613019 2 2.048606

WindvsLee 2.010220 1 1.417822

Rugosity.t. 1.479861 1 1.216495

Hum.Reef 1.189792 1 1.090776

#Run vif again without Depth as is show values > 3

LFvsGrper.Biom.glm1= glm(LF.Count ~ Grouper.Biom + Predators

+ Time + Protection + Habitat + WindvsLee + Rugosity.t. + Hum.Reef,

data=fish, offset = LogArea, family=poisson)

summary(LFvsGrper.Biom.glm1)

Call:

glm(formula = LF.Count ~ Grouper.Biom + Predators + Time + Protection +

Habitat + WindvsLee + Rugosity.t. + Hum.Reef, family = poisson,

data = fish, offset = LogArea)

Deviance Residuals:

Min 1Q Median 3Q Max

-5.436 -1.326 -1.042 0.618 8.094

Coefficients:

Estimate Std. Error z value Pr(>|z|)

(Intercept) -2.517e+00 2.517e-01 -10.000 < 2e-16 ***

Grouper.Biom 1.347e-02 2.036e-03 6.614 3.75e-11 ***

Predators -8.991e-04 4.271e-04 -2.105 0.0353 *

Time 6.586e-02 5.694e-02 1.157 0.2474

Protectiony -6.576e-01 1.635e-01 -4.023 5.75e-05 ***

HabitatS&G -3.550e+00 1.435e-01 -24.734 < 2e-16 ***

HabitatSlope -3.523e+00 1.470e-01 -23.973 < 2e-16 ***

WindvsLeeWindward -9.182e-01 1.572e-01 -5.840 5.23e-09 ***

Rugosity.t. 6.411e-02 4.333e-02 1.480 0.1390

Hum.Reef 5.256e-05 1.042e-05 5.044 4.56e-07 ***

---

Signif. codes: 0 ‘***’ 0.001 ‘**’ 0.01 ‘*’ 0.05 ‘.’ 0.1 ‘ ’ 1

(Dispersion parameter for poisson family taken to be 1)

Null deviance: 8263.5 on 362 degrees of freedom

Residual deviance: 1525.0 on 353 degrees of freedom

AIC: 2253.5

Number of Fisher Scoring iterations: 7

vif(LFvsGrper.Biom.glm1)

GVIF Df GVIF^(1/(2*Df))

Grouper.Biom 1.306581 1 1.143058

Predators 1.388809 1 1.178477

Time 1.843376 1 1.357710

Protection 2.515460 1 1.586020

Habitat 5.427136 2 1.526310

WindvsLee 2.057991 1 1.434570

Rugosity.t. 1.549107 1 1.244631

Hum.Reef 1.392757 1 1.180151

# The differences in the AIC are small but with Depth include is lower

anova(LFvsGrper.Biom.glmp, LFvsGrper.Biom.glm1)

Analysis of Deviance Table

Model 1: LF.Count ~ Grouper.Biom + Predators + Time + Depth + Protection +

Habitat + WindvsLee + Rugosity.t. + Hum.Reef

Model 2: LF.Count ~ Grouper.Biom + Predators + Time + Protection + Habitat +

WindvsLee + Rugosity.t. + Hum.Reef

Resid. Df Resid. Dev Df Deviance

1 352 1518.5

2 353 1525.0 -1 -6.452

AIC(LFvsGrper.Biom.glmp, LFvsGrper.Biom.glm1)

df AIC

LFvsGrper.Biom.glmp 11 2249.020

LFvsGrper.Biom.glm1 10 2253.472

#Rename LF biomass variable to make easier to work with

LF.Biom <- LF.Biom..g100m2.

#Calculate correlation values between LF biomass and abundance

cor.test(LF.Biom,LF.Abund); plot (LF.Biom,LF.Abund)

Pearson's product-moment correlation

data: LF.Biom and LF.Abund

t = 64.965, df = 361, p-value < 2.2e-16

alternative hypothesis: true correlation is not equal to 0

95 percent confidence interval:

0.9507939 0.9671752

sample estimates:

cor

0.9597937


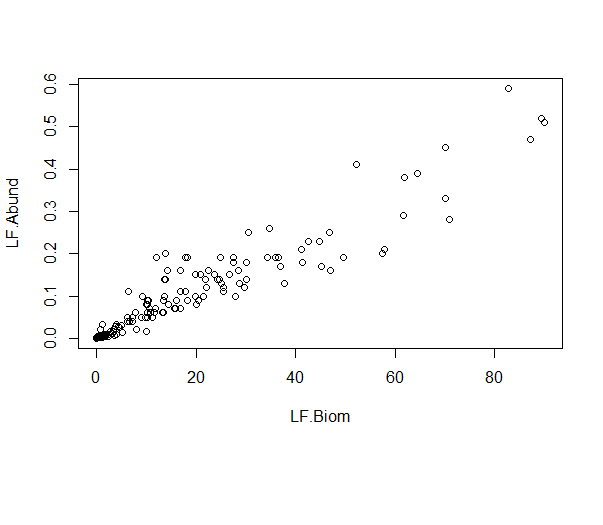


#Use the same model as in Hackerott et al 2013 to make things comparable

#Add rugosity and humans/reef areas to the model

#Scale numerical variables to make easy to visualize factors effect

# Run a glmm with ADMB and a negative binomial distribution as works better than poisson here

# Also the glmmADMB account for overdispersion on the LF data

# Use Lionfish counts (discrete dist) and add log area of survey as offset

# set zeroinflation to TRUE

# Load first glmmADMB library

library(glmmADMB)

#Run a null model with structure first

LFvsGrper.Biom.out0= glmmadmb(LF.Count ~ 1 + (1|Site.Code),

data = fish,

zeroInflation=TRUE,

#admb.opts=admbControl(shess=FALSE,noinit=F),

family= "nbinom")

summary(LFvsGrper.Biom.out0)

Call:

glmmadmb(formula = LF.Count ~ 1 + (1 | Site.Code), data = fish,

family = "nbinom", zeroInflation = TRUE)

AIC: 1487.2

Coefficients:

Estimate Std. Error z value Pr(>|z|)

(Intercept) 0.488 0.270 1.81 0.07 .

---

Signif. codes: 0 ‘***’ 0.001 ‘**’ 0.01 ‘*’ 0.05 ‘.’ 0.1 ‘ ’ 1

Number of observations: total=363, Site.Code=71

Random effect variance(s):

Group=Site.Code

Variance StdDev

(Intercept) 4.418 2.102

Negative binomial dispersion parameter: 403.43 (std. err.: 0.51035)

Zero-inflation: 0.084676 (std. err.: 0.026548 )

Log-likelihood: -739.584

#Run a null model with structure and Poisson instead and compare AIC

LFvsGrper.Biom.out0p= glmmadmb(LF.Count ~ 1 + (1|Site.Code),

data = fish,

zeroInflation=T,

#admb.opts=admbControl(shess=FALSE,noinit=F),

family= "poisson")

summary(LFvsGrper.Biom.out0p)

Call:

glmmadmb(formula = LF.Count ~ 1 + (1 | Site.Code), data = fish,

family = "poisson", zeroInflation = T)

AIC: 2300

Coefficients:

Estimate Std. Error z value Pr(>|z|)

(Intercept) 0.0527 0.2844 0.19 0.85

Number of observations: total=363, Site.Code=71

Random effect variance(s):

Group=Site.Code

Variance StdDev

(Intercept) 4.25 2.062

Zero-inflation: 0.051061 (std. err.: 0.029928 )

Log-likelihood: -1147.01

AIC(LFvsGrper.Biom.out0, LFvsGrper.Biom.out0p)

df AIC

LFvsGrper.Biom.out0 4 1487.168

LFvsGrper.Biom.out0p 3 2300.020

#AIC for binomial is 1487 and for poisson is 2300, stick with nbinom

#Run the intercept model with a negative binomial (log link) type 1

LFvsGrper.Biom.null0= glmmadmb(LF.Count ~ 1 + (1|Site.Code),

data = fish,

zeroInflation=T,verbose=T,

admb.opts=admbControl(shess=TRUE,noinit=FALSE),

family= "nbinom1")

summary(LFvsGrper.Biom.null0)

Call:

glmmadmb(formula = LF.Count ~ 1 + (1 | Site.Code), data = fish,

family = "nbinom1", zeroInflation = T, admb.opts = admbControl(shess = TRUE, noinit = FALSE), verbose = T)

AIC: 1475.4

Coefficients:

Estimate Std. Error z value Pr(>|z|)

(Intercept) 0.228 0.291 0.78 0.43

Number of observations: total=363, Site.Code=71

Random effect variance(s):

Group=Site.Code

Variance StdDev

(Intercept) 4.521 2.126

Negative binomial dispersion parameter: 1.7684 (std. err.: 0.19832)

Zero-inflation: 0.013701 (std. err.: 0.013083 )

Log-likelihood: -733.677

AIC(LFvsGrper.Biom.out0,LFvsGrper.Biom.null0)

df AIC

LFvsGrper.Biom.out0 4 1487.168

LFvsGrper.Biom.null0 4 1475.354

#Calculate a deviance table those models fitting are different

anova(LFvsGrper.Biom.out0,LFvsGrper.Biom.null0)

Analysis of Deviance Table

Model 1: LF.Count ~ 1

Model 2: LF.Count ~ 1

NoPar LogLik Df Deviance Pr(>Chi)

1 4 -739.58

2 4 -733.68 0 11.814 < 2.2e-16 ***

---

Signif. codes: 0 ‘***’ 0.001 ‘**’ 0.01 ‘*’ 0.05 ‘.’ 0.1 ‘ ’ 1

#The negative binomial distribution type 1 and log link is a little better

# AIC for type 2 1487 and type 1 1475

# Use nbinom type 1 for the full model

# Now chose the structure of the random effects

# Try sites nested within region

LFvsGrper.Biom.null= glmmadmb(LF.Count ~ 1

+ (1|Region/Site.Code),

data = fish,

zeroInflation=T,verbose=T, admb.opts=admbControl(shess=TRUE,noinit=FALSE),

family= "nbinom1")

AIC(LFvsGrper.Biom.null, LFvsGrper.Biom.null0)

df AIC

LFvsGrper.Biom.null 5 1270.546

LFvsGrper.Biom.null0 4 1475.354

anova(LFvsGrper.Biom.null, LFvsGrper.Biom.null0)

Analysis of Deviance Table

Model 1: LF.Count ~ 1

Model 2: LF.Count ~ 1

NoPar LogLik Df Deviance Pr(>Chi)

1 4 -733.68

2 5 -630.27 1 206.81 < 2.2e-16 ***

---

Signif. codes: 0 ‘***’ 0.001 ‘**’ 0.01 ‘*’ 0.05 ‘.’ 0.1 ‘ ’ 1

# Looks like sites nested within region is a better random effect structure

#Check distribution of model residuals

#Build function first

plot.glmer<-

function(mer.fit,type="pearson",overdispersion.term=NULL)

{

require(AICcmodavg)

if(is.null(overdispersion.term))

{

Fitted<-fitted(mer.fit)

Residuals=resid(mer.fit,type)

} else

{

response<-model.frame(mer.fit)[[1]]

od.ranef<-lme4::ranef(mer.fit)[[overdispersion.term]][[1]]

if(length(response)!=length(od.ranef) || fam.link.mer(mer.fit)$family!="nbinom1" || fam.link.mer(mer.fit)$link!="log")

stop("Model is not lognormal-Poisson. Cannot use overdispersion term.")

Fitted<-exp(log(fitted(mer.fit))-od.ranef)

Residuals<-(response - Fitted)/sqrt(Fitted+(Fitted^2)*c(exp(lme4::VarCorr(mer.fit)[[overdispersion.term]])-1))

}

plot.data<-data.frame(Fitted=Fitted,Residuals=Residuals)

plot.data$loess.line<-predict(loess(Residuals~Fitted,data=plot.data))

plot.data<-plot.data[order(plot.data$Fitted),]

plot(plot.data[,c("Fitted","Residuals")])

abline(h=0)

points(plot.data[,c("Fitted","loess.line")],type="l",col="red")

hist(plot.data$Residuals,xlab="Residuals",main="")

}

plot.glmer(LFvsGrper.Biom.null)


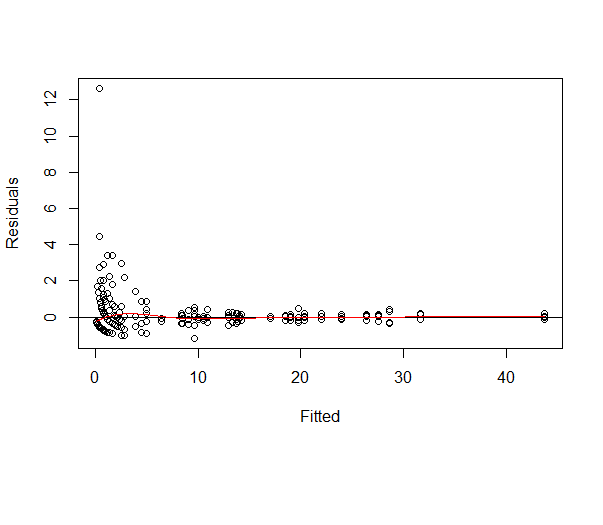


#Figure Residuals vs Fitted of the intercept model

#check if the model with zero inflation = FALSE is better

LFvsGrper.Biom.outt1.ZIf= glmmadmb(LF.Count ~ 1 + (1|Region/Site.Code),

data = fish,

zeroInflation=FALSE,

admb.opts=admbControl(shess=TRUE,noinit=F),

family= "nbinom1")

summary(LFvsGrper.Biom.outt1.ZIf)

Call:

glmmadmb(formula = LF.Count ~ 1 + (1 | Region/Site.Code), data = fish,

family = "nbinom1", zeroInflation = FALSE, admb.opts = admbControl(shess = TRUE, noinit = F))

AIC: 1270.8

Coefficients:

Estimate Std. Error z value Pr(>|z|)

(Intercept) -0.628 0.577 -1.09 0.28

Number of observations: total=363, Region=11, Region:Site.Code=71

Random effect variance(s):

Group=Region

Variance StdDev

(Intercept) 2.851 1.688

Group=Region:Site.Code

Variance StdDev

(Intercept) 0.457 0.676

Negative binomial dispersion parameter: 1.679 (std. err.: 0.17504)

Log-likelihood: -631.406

AIC(LFvsGrper.Biom.outt1.ZIf,LFvsGrper.Biom.null)

df AIC

LFvsGrper.Biom.outt1.ZIf 4 1270.812

LFvsGrper.Biom.null 5 1270.546

#Calculate deviance between models since AIC is very similar

anova(LFvsGrper.Biom.outt1.ZIf,LFvsGrper.Biom.null)

Analysis of Deviance Table

Model 1: LF.Count ~ 1

Model 2: LF.Count ~ 1

NoPar LogLik Df Deviance Pr(>Chi)

1 4 -631.41

2 5 -630.27 1 2.266 0.1322

#With zero inflation or without the AIC are similar. However this might change when the predictor variables are in

#Run model with all variables for LF Counts and negative binomial type 1

#Run also a MCMC for PostHoc comparisons (this might take a while)

detach(package:lme4)

detach(package:nlme)

library(glmmADMB)

#Run the intercept model with the offset for comparison with the full model

LFvsGrper.Biom.nulloffset= glmmadmb(LF.Count ~ 1

+ offset(scale(LogArea))

+ (1|Region/Site.Code), mcmc=F, mcmc.opts=mcmcControl(mcmc=1000),

verbose=T, corStruct="diag",

data = fish,

zeroInflation=TRUE,

admb.opts=admbControl(shess=TRUE,noinit=FALSE),

family= "nbinom1")

summary(LFvsGrper.Biom.nulloffset)

Call:

glmmadmb(formula = LF.Count ~ 1 + offset(scale(LogArea)) + (1 |

Region/Site.Code), data = fish, family = "nbinom1", corStruct = "diag",

zeroInflation = TRUE, admb.opts = admbControl(shess = TRUE,

noinit = FALSE), mcmc = F, mcmc.opts = mcmcControl(mcmc = 1000),

verbose = T)

AIC: 1337.5

Coefficients:

Estimate Std. Error z value Pr(>|z|)

(Intercept) -1.165 0.768 -1.52 0.13

Number of observations: total=363, Region=11, Region:Site.Code=71

Random effect variance(s):

Group=Region

Variance StdDev

(Intercept) 5.259 2.293

Group=Region:Site.Code

Variance StdDev

(Intercept) 0.7785 0.8823

Negative binomial dispersion parameter: 1.7563 (std. err.: 0.16917)

Zero-inflation: 0.0085216 (std. err.: 0.0088282 )

Log-likelihood: -663.746

#Run the full model

LFvsGrper.Biom.full= glmmadmb(LF.Count ~

Habitat + WindvsLee + Protection

+ scale(Depth) + scale(Time) + scale(Rugosity.t.) + scale(Hum.Reef)

+ scale(Predators) + scale(log(Grouper.Biom+1))

+ offset(scale(LogArea))

+ (1|Region/Site.Code),

mcmc=T, mcmc.opts=mcmcControl(mcmc=1000),

verbose=T, corStruct="diag",

data = fish,

zeroInflation=TRUE,

admb.opts=admbControl(shess=TRUE,noinit=FALSE),

family= "nbinom1")

summary(LFvsGrper.Biom.full)

Call:

glmmadmb(formula = LF.Count ~ Habitat + WindvsLee + Protection +

scale(Depth) + scale(Time) + scale(Rugosity.t.) + scale(Hum.Reef) +

scale(Predators) + scale(log(Grouper.Biom + 1)) + offset(scale(LogArea)) +

(1 | Region/Site.Code), data = fish, family = "nbinom1",

corStruct = "diag", zeroInflation = TRUE, admb.opts = admbControl(shess = TRUE,

noinit = FALSE), mcmc = T, mcmc.opts = mcmcControl(mcmc = 1000),

verbose = T)

AIC: 1334.3

Coefficients:

Estimate Std. Error z value Pr(>|z|)

(Intercept) 4.07638 1.27310 3.20 0.0014 **

HabitatS&G -4.48701 1.56160 -2.87 0.0041 **

HabitatSlope -4.27408 1.44060 -2.97 0.0030 **

WindvsLeeWindward -1.38936 0.71772 -1.94 0.0529 .

Protectiony -1.35068 0.55604 -2.43 0.0151 *

scale(Depth) -0.08408 0.33838 -0.25 0.8038

scale(Time) 0.33751 0.37801 0.89 0.3719

scale(Rugosity.t.) 0.00591 0.08434 0.07 0.9441

scale(Hum.Reef) 0.07290 0.10960 0.67 0.5059

scale(Predators) -0.10706 0.10199 -1.05 0.2939

scale(log(Grouper.Biom + 1)) 0.01343 0.04754 0.28 0.7776

---

Signif. codes: 0 ‘***’ 0.001 ‘**’ 0.01 ‘*’ 0.05 ‘.’ 0.1 ‘ ’ 1

Number of observations: total=363, Region=11, Region:Site.Code=71

Random effect variance(s):

Group=Region

Variance StdDev

(Intercept) 1.327 1.152

Group=Region:Site.Code

Variance StdDev

(Intercept) 0.6669 0.8167

Negative binomial dispersion parameter: 1.7341 (std. err.: 0.1669)

Zero-inflation: 0.0086228 (std. err.: 0.009031 )

Log-likelihood: -652.132

#Compare models

AIC(LFvsGrper.Biom.full, LFvsGrper.Biom.nulloffset)

df AIC

LFvsGrper.Biom.full 15 1334.264

LFvsGrper.Biom.nulloffset 5 1337.492

anova(LFvsGrper.Biom.full, LFvsGrper.Biom.nulloffset)

Analysis of Deviance Table

Model 1: LF.Count ~ 1 + offset(scale(LogArea))

Model 2: LF.Count ~ Habitat + WindvsLee + Protection + scale(Depth) + scale(Time) + scale(Rugosity.t.) + scale(Hum.Reef) + scale(Predators) + scale(log(Grouper.Biom + 1)) + offset(scale(LogArea))

NoPar LogLik Df Deviance Pr(>Chi)

1 5 -663.75

2 15 -652.13 10 23.228 0.009936 **

---

Signif. codes: 0 ‘***’ 0.001 ‘**’ 0.01 ‘*’ 0.05 ‘.’ 0.1 ‘ ’ 1

#Convert the MCMC chain to a mcmc object which the coda package can handle:

library(coda)

m <- as.mcmc(LFvsGrper.Biom.full$mcmc)

#Look at the trace plots

install.packages("scapeMCMC")

library(scapeMCMC)

plotTrace(m)

#see other page…


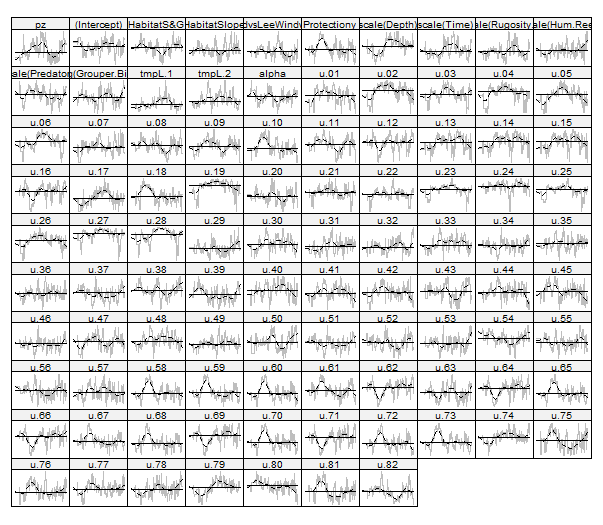


##The Geweke diagnostic gives Z scores for each variable for a comparison between the first 10% and last 50% of the chain

gg <- geweke.diag(m)

summary(2*pnorm(abs(gg$z),lower.tail=FALSE))

Min. 1st Qu. Median Mean 3rd Qu. Max.

0.0001483 0.0316800 0.2578000 0.3316000 0.6396000 0.9713000

#The most frequently used diagnostic, Gelman-Rubin (gelman.diag), requires multiple chains. The full set of diagnostic functions available in coda is:

## [1] autocorr.diag gelman.diag geweke.diag heidel.diag raftery.diag

# effectiveSize gives the effective length of the chain for each variable, i.e. the number of samples corrected for autocorrelation:

range(effectiveSize(m))

[1] 7.334345 33.479108

# HPDinterval gives the highest posterior density (credible interval):

head(HPDinterval(m))

lower upper

pz 0.003571724 0.03382066

(Intercept) 1.480278900 5.32406265

HabitatS&G -7.572696048 -2.51577889

HabitatSlope -5.748568618 -1.82659370

WindvsLeeWindward -2.602530904 -0.20973529

Protectiony -2.198550576 0.03624813

# Or inferences based on the quantiles instead:

head(t(apply(m,2,quantile,c(0.025,0.975))))

2.5% 97.5%

pz 0.003714645 0.034850496

(Intercept) 1.159307085 5.064113270

HabitatS&G -7.503009398 -2.254868334

HabitatSlope -5.713138447 -1.641416360

WindvsLeeWindward -2.471194167 -0.004532558

Protectiony -2.316347129 -0.007886751

## Export Summary Table to Word a doc

capture.output(summary(LFvsGrper.Biom.full),file="glmmADMDresults.doc")

## Check residuals vs fitted

plot(residuals(LFvsGrper.Biom.full)~fitted(LFvsGrper.Biom.full))

points(predict(loess(residuals(LFvsGrper.Biom.full)~

fitted(LFvsGrper.Biom.full))), type="l", col=2)

abline(h=0,lty=2)


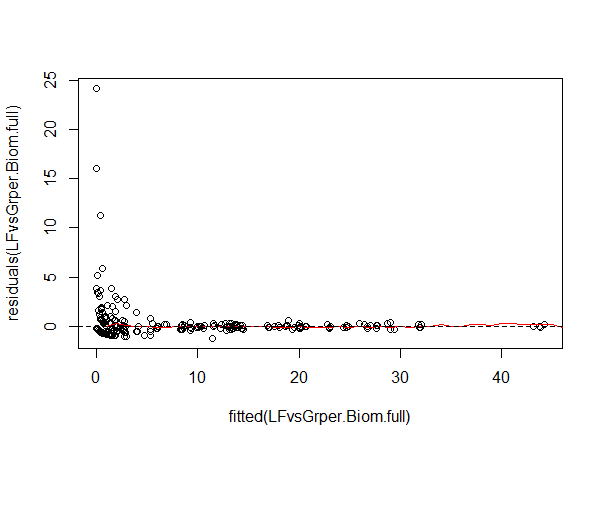


## Calculate Pseudo R squared

## Use the function Jarred Byrners wrote…

r2.corr.mer <- function(m) {

lmfit <- lm(model.response(model.frame(m)) ~ fitted(m))

summary(lmfit)$r.squared }

#Run the function with the results of the full and null model

r2.corr.mer (LFvsGrper.Biom.full)

[1] 0.9458507

# coefficiente quite high 0.9464561 (check code)

r2.corr.mer (LFvsGrper.Biom.null)

[1] 0.9498226

m = LFvsGrper.Biom.full

1-var(residuals(m))/(var(model.response(model.frame(m))))

[,1]

[1,] 0.9593797

#Plot estimates

#Require coefplot2 package from R-Forge or Ben Bolker directly

library(coefplot2)

coefplot2(LFvsGrper.Biom.full)


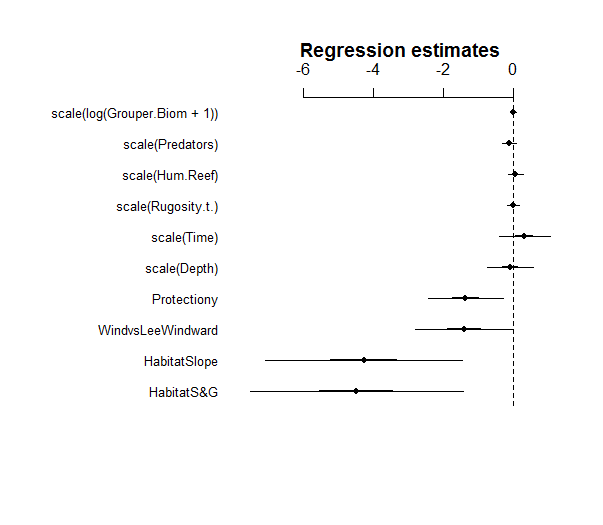


##Test for overdispersion on the glmmADMB model residuals

## Build function

overdisp_fun <- function(model) {

## number of variance parameters in

## an n-by-n variance-covariance matrix

vpars <- function(m) {

nrow(m)*(nrow(m)+1)/2

}

model.df <- sum(sapply(VarCorr(model),vpars))+length(fixef(model))

rdf <- nrow(model.frame(model))-model.df

rp <- residuals(model,type="pearson")

Pearson.chisq <- sum(rp^2)

prat <- Pearson.chisq/rdf

pval <- pchisq(Pearson.chisq, df=rdf, lower.tail=FALSE)

c(chisq=Pearson.chisq,ratio=prat,rdf=rdf,p=pval)

}

#check for overdispersion in the model residuals

overdisp_fun (LFvsGrper.Biom.full)

chisq ratio rdf p

1.236366e+03 3.532473e+00 3.500000e+02 3.282660e-99

##Run a glmm with negative binomial distribution and log link to see if we get the same results

##Detach package glmmADMB as the use the same VarCorr

detach(package:glmmADMB)

#Load lme4 and nlme

library (lme4)

library (nlme)

#Run Null model first with Poisson distribution

LFvsGrper.Biom.glmer.null= glmer(LF.Count ~ 1

+ offset(scale(LogArea)) + (1|Site.Code),

data = fish, nAGQ = 2,

family= poisson(link="log"))

summary(LFvsGrper.Biom.glmer.null)

Generalized linear mixed model fit by maximum likelihood ['glmerMod']

Family: poisson ( log )

Formula: LF.Count ~ 1 + offset(scale(LogArea)) + (1 | Site.Code)

Data: fish

AIC BIC logLik deviance

818.6113 826.4001 -407.3056 814.6113

Random effects:

Groups Name Variance Std.Dev.

Site.Code (Intercept) 10.17 3.188

Number of obs: 363, groups: Site.Code, 71

Fixed effects:

Estimate Std. Error z value Pr(>|z|)

(Intercept) 0.4543 0.3879 1.171 0.241

#Run full model with Poisson distribution

LFvsGrper.Biom.glmer= glmer(LF.Count ~ scale(log(Grouper.Biom+1)) + scale(Predators)

+ Habitat + Protection + WindvsLee + scale(Depth)+ scale(Time)+ scale(Rugosity.t.)+ scale(Hum.Reef)

+ offset(scale(LogArea)) + (1|Site.Code),

data = fish, nAGQ = 2,

family= poisson(link="log"))

summary(LFvsGrper.Biom.glmer)

Generalized linear mixed model fit by maximum likelihood ['glmerMod']

Family: poisson ( log )

Formula: LF.Count ~ scale(log(Grouper.Biom + 1)) + scale(Predators) + Habitat + Protection + WindvsLee + scale(Depth) + scale(Time) + scale(Rugosity.t.) + scale(Hum.Reef) + offset(scale(LogArea)) + (1 | Site.Code)

Data: fish

AIC BIC logLik deviance

715.3343 762.0671 -345.6671 691.3343

Random effects:

Groups Name Variance Std.Dev.

Site.Code (Intercept) 1.174 1.083

Number of obs: 363, groups: Site.Code, 71

Fixed effects:

Estimate Std. Error z value Pr(>|z|)

(Intercept) 4.15021 0.47137 8.805 < 2e-16 ***

scale(log(Grouper.Biom + 1)) 0.02206 0.03617 0.610 0.5420

scale(Predators) -0.04294 0.07002 -0.613 0.5397

HabitatS&G -4.17804 0.79802 -5.236 1.65e-07 ***

HabitatSlope -4.50883 0.66369 -6.794 1.09e-11 ***

Protectiony -1.11992 0.47411 -2.362 0.0182 *

WindvsLeeWindward -1.41119 0.45436 -3.106 0.0019 **

scale(Depth) 0.04033 0.27384 0.147 0.8829

scale(Time) 0.02465 0.19296 0.128 0.8983

scale(Rugosity.t.) -0.04876 0.06690 -0.729 0.4661

scale(Hum.Reef) 0.12882 0.13756 0.936 0.3490

---

Signif. codes: 0 ‘***’ 0.001 ‘**’ 0.01 ‘*’ 0.05 ‘.’ 0.1 ‘ ’ 1

Correlation of Fixed Effects:

(Intr) s((G+1 scl(P) HbtS&G HbttSl Prtctn WndvLW scl(D) scl(T) s(R..)

sc((G.B+1)) 0.021

scl(Prdtrs) 0.029 -0.143

HabitatS&G -0.851 -0.002 -0.001

HabitatSlop -0.716 -0.001 -0.051 0.759

Protectiony -0.025 -0.067 -0.045 -0.298 -0.346

WndvsLWndwr 0.100 -0.001 0.012 -0.477 -0.327 0.390

scale(Dpth) 0.874 0.055 -0.006 -0.784 -0.591 -0.075 0.064

scale(Time) 0.255 -0.005 -0.017 -0.445 -0.428 0.435 0.389 0.164

scl(Rgst..) 0.066 0.030 -0.213 -0.037 -0.083 -0.078 0.037 -0.014 0.076

scal(Hm.Rf) -0.156 -0.002 -0.003 0.047 0.098 0.109 0.154 -0.159 -0.141 -0.008

#Compare models

AIC(LFvsGrper.Biom.glmer.null, LFvsGrper.Biom.glmer)

df AIC

LFvsGrper.Biom.glmer.null 2 818.6113

LFvsGrper.Biom.glmer 12 715.3343

#Check model residuals (residuals vs fitted)

plot(LFvsGrper.Biom.glmer)


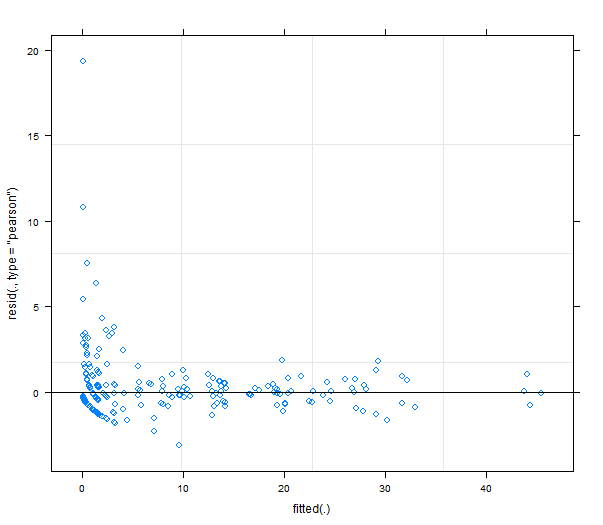


#Run null model with negative binomial distribution

LFvsGrper.Biom.glmer.nb.null= glmer.nb(LF.Count ~ 1 + offset(scale(LogArea)) + (1|Region/Site.Code), data = fish)

summary(LFvsGrper.Biom.glmer.nb.null)

Generalized linear mixed model fit by maximum likelihood ['glmerMod']

Family: Negative Binomial(210.7156) ( log )

Formula: LF.Count ~ 1 + offset(scale(LogArea)) + (1 | Region/Site.Code)

Data: ..2

AIC BIC logLik deviance

1419.6385 1435.2161 -705.8192 1411.6385

Random effects:

Groups Name Variance Std.Dev.

Site.Code:Region (Intercept) 2.494 1.579

Region (Intercept) 14.383 3.792

Residual 2.624 1.620

Number of obs: 363, groups: Site.Code:Region, 71; Region, 11

Fixed effects:

Estimate Std. Error t value Pr(>|z|)

(Intercept) -1.193 1.227 -0.972 0.331

#Run full model with negative binomial distribution

LFvsGrper.Biom.glmer.nb= glmer.nb(LF.Count ~ Habitat + WindvsLee + Protection

+ scale(Depth) + scale(Time) + scale(Rugosity.t.)+scale(Hum.Reef)

+ scale(log(Predators+1)) + scale(log(Grouper.Biom+1))

+ offset(log(Area4LF)) + (1|Region/Site.Code), data = fish)

summary(LFvsGrper.Biom.glmer.nb)

Generalized linear mixed model fit by maximum likelihood ['glmerMod']

Family: Negative Binomial(627.2293) ( log )

Formula: LF.Count ~ Habitat + WindvsLee + Protection + scale(Depth) + scale(Time) + scale(Rugosity.t.) + scale(Hum.Reef) + scale(log(Predators + 1)) + scale(log(Grouper.Biom + 1)) + offset(log(Area4LF)) + (1 | Region/Site.Code)

Data: ..2

AIC BIC logLik deviance

1369.4303 1423.9519 -670.7151 1341.4303

Random effects:

Groups Name Variance Std.Dev.

Site.Code:Region (Intercept) 1.101 1.049

Region (Intercept) 2.300 1.517

Residual 1.717 1.310

Number of obs: 363, groups: Site.Code:Region, 71; Region, 11

Fixed effects:

Estimate Std. Error t value Pr(>|z|)

(Intercept) -2.137982 1.623073 -1.317 0.1878

HabitatS&G -3.793741 1.929422 -1.966 0.0493 *

HabitatSlope -3.429744 1.824025 -1.880 0.0601 .

WindvsLeeWindward -1.677717 0.861382 -1.948 0.0515 .

Protectiony -1.483753 0.647989 -2.290 0.0220 *

scale(Depth) -0.078244 0.347050 -0.226 0.8216

scale(Time) 0.226548 0.477120 0.475 0.6349

scale(Rugosity.t.) -0.040434 0.086410 -0.468 0.6398

scale(Hum.Reef) 0.085699 0.137357 0.624 0.5327

scale(log(Predators + 1)) -0.008941 0.041666 -0.215 0.8301

scale(log(Grouper.Biom + 1)) 0.010804 0.047886 0.226 0.8215

---

Signif. codes: 0 ‘***’ 0.001 ‘**’ 0.01 ‘*’ 0.05 ‘.’ 0.1 ‘ ’ 1

Correlation of Fixed Effects:

(Intr) HbtS&G HbttSl WndvLW Prtctn scl(D) scl(T) s(R..) s(H.R) s((P+1

HabitatS&G -0.889

HabitatSlop -0.889 0.889

WndvsLWndwr 0.012 -0.265 -0.162

Protectiony 0.005 -0.195 -0.173 0.376

scale(Dpth) 0.320 -0.409 -0.267 -0.004 -0.035

scale(Time) 0.099 -0.109 -0.138 0.116 0.197 0.062

scl(Rgst..) 0.025 -0.059 -0.053 0.140 0.006 -0.023 0.049

scal(Hm.Rf) -0.061 0.065 0.054 0.018 0.020 -0.170 -0.067 -0.039

scl(l(P+1)) 0.011 -0.014 -0.022 0.008 0.015 -0.011 0.005 -0.121 -0.009

sc((G.B+1)) 0.007 -0.010 -0.005 0.030 -0.042 0.054 -0.003 0.025 -0.006 -0.146

AIC(LFvsGrper.Biom.glmer.nb.null, LFvsGrper.Biom.glmer.nb)

df AIC

LFvsGrper.Biom.glmer.nb.null 4 1419.638

LFvsGrper.Biom.glmer.nb 14 1369.430

#Plot model coefficients

coefplot2 (LFvsGrper.Biom.glmer.nb)


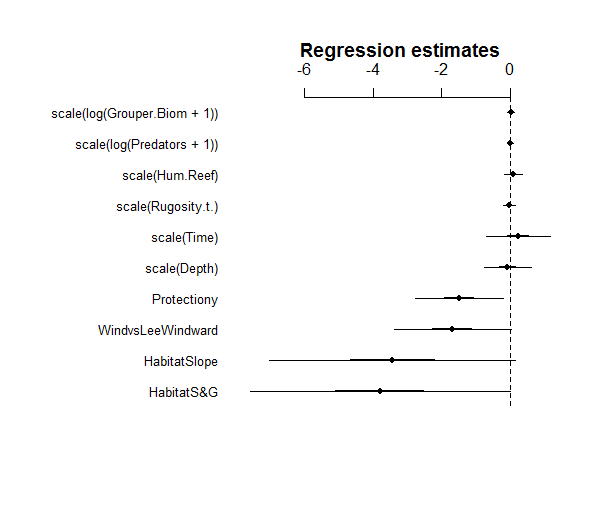


#Check model residuals for glmer.nb

plot(LFvsGrper.Biom.glmer.nb)
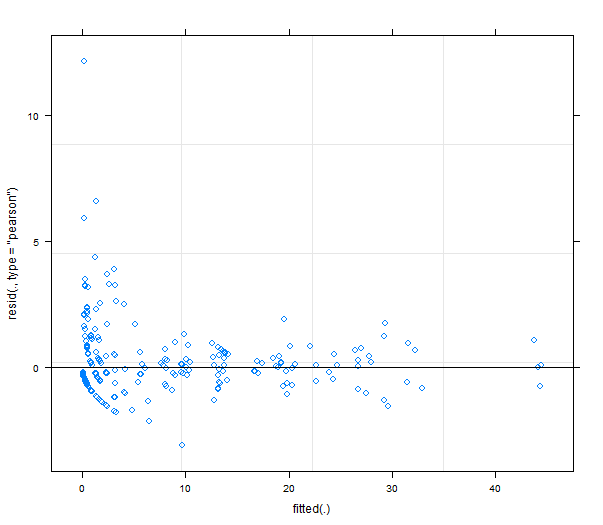


#In the end the glmer.nb give the same ecological results as the glmmADMB

##Check Autocorrelation for LF and grouper since we have 71 sites across the Caribbean

##Use Moran's I simmilarity spline correlograms for LF Biomass and grouper Biomass

##Check for autocorrelation on the model residuals

library(ncf)

CorRes.glmer <- spline.correlog(Longitude, Latitude, residuals(LFvsGrper.Biom.glmer), resamp=100,latlon=TRUE, quiet=TRUE, filter=TRUE)

plot(CorRes.glmer)


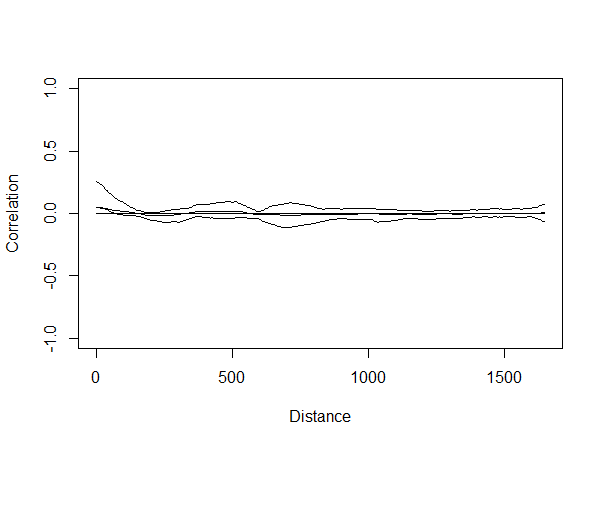


CorRes.glmer.nb <- spline.correlog(Longitude, Latitude, residuals(LFvsGrper.Biom.glmer.nb), resamp=100,latlon=TRUE, quiet=TRUE, filter=TRUE)

plot(CorRes.glmer.nb)


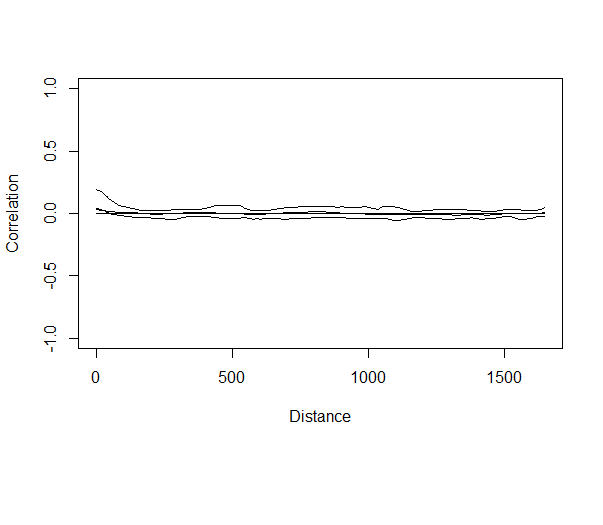


CorRes.glmmADMB <- spline.correlog(Longitude, Latitude, as.vector(residuals(LFvsGrper.Biom.full)), resamp=100,latlon=TRUE, quiet=TRUE, filter=TRUE, na.rm=T)

plot(CorRes.glmmADMB)


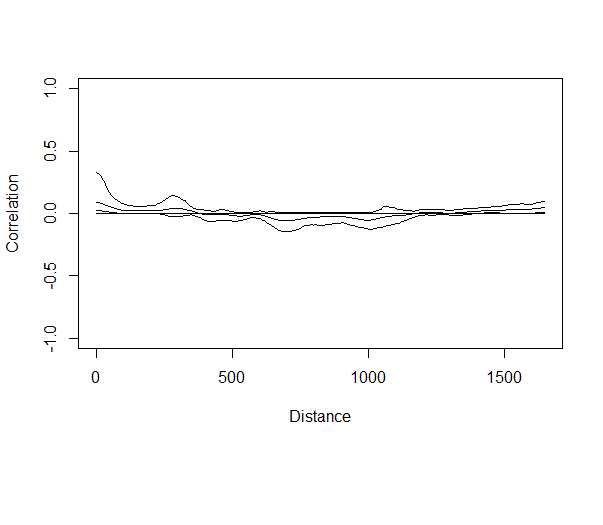


CorLF <- spline.correlog(Longitude, Latitude, fish$LF.Biom, resamp=100, latlon=TRUE, quiet=TRUE)

CorGrouper <- spline.correlog(Longitude, Latitude, Grouper.Biom, resamp=100, latlon=TRUE, quiet=TRUE)

#Plot Correlograms

plot (CorLF)


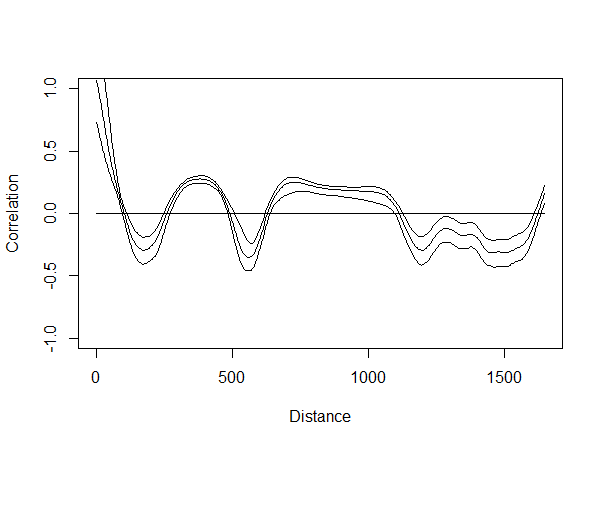


plot(CorGrouper)


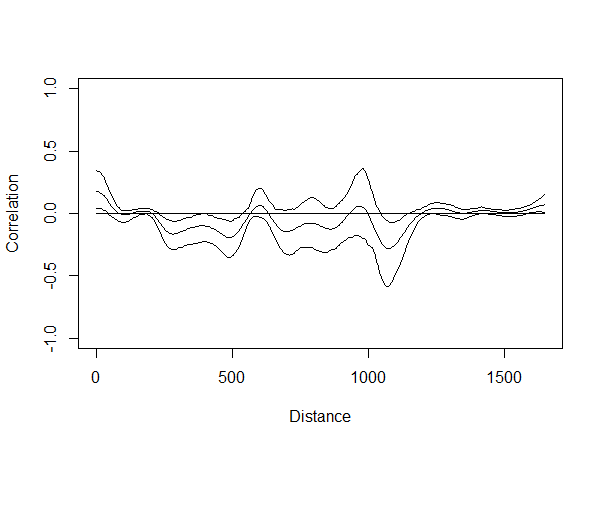


#Check for autocorrelation on the model residuals since the raw data is autocorrelated

CorRes <- spline.correlog(Longitude, Latitude, as.vector(residuals(LFvsGrper.Biom.null)), resamp=100,latlon=TRUE, quiet=TRUE, filter=TRUE)

plot(CorRes)


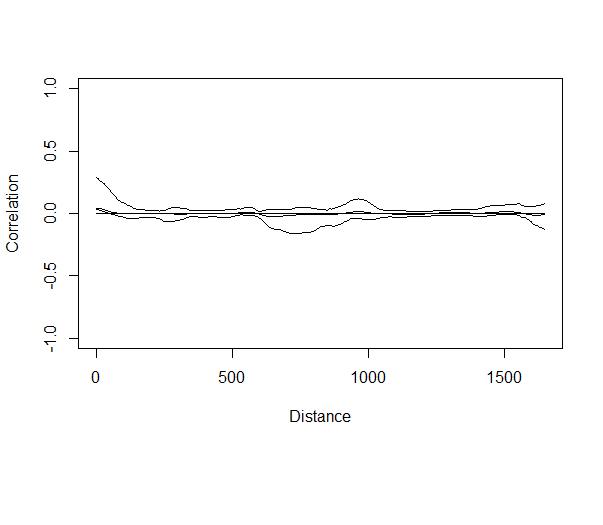


# The spatial structure of the glmer and glmmADMB accomodates nicely the spatial autocorrelation :)

###FIGURE, Plot all correlograms together to compare and save as supplemental figure for the paper

png("./CorFish.png", width=3, height=6, units="in", res=600)

par(mfrow=c(3,1), mai=c(0.5,1,0.2,0.2), mgp=c(0.5,0.6,0), mar=c(1.5,2.5,1,0.2), oma=c(2.5,1.5,0,0.5), bg="white", fg="black", cex.axis=1.05)

plot(CorLF,text=F)

legend("topright", "Lionfish", bty="n", cex=1.4)

plot(CorGrouper, text=F)

legend("topright", "Grouper", bty="n", cex=1.4)

mtext("Moran's I Similarity", side=2, adj=0.5, line=2.5, cex=1)

plot(CorRes.glmmADMB, text=F)

legend("topright", "glmmADMB Model residuals", bty="n", cex=1.4)

mtext("distance (km)", side=1, adj=0.5, line=2, cex=0.9)

dev.off()

##### Mantel's Test to test for overal autocorrelation (numerical representation)

library(ade4)

library(ecodist)

#Generate distance matrices

Site.dists <- dist(cbind(fish$Longitude, fish$Latitude))

LF.dists <- dist(fish$LF.Abund)

LF.distsresiduals <- dist(residuals(LFvsGrper.Biom.full))

#Mantel Test

mantel.results=mantel.rtest(LF.dists, Site.dists, nrepet=999)

#Plot Mantel Test

mantel1=mgram(LF.dists, Site.dists)

mantel2=mgram(LF.distsresiduals, Site.dists)

par(mfrow=c(1,2))

plot(mantel1, ylim=c(-1,1));abline(0,0, lty=2,col="grey")

plot(mantel2, ylim=c(-1,1));abline(0,0, lty=2,col="grey")


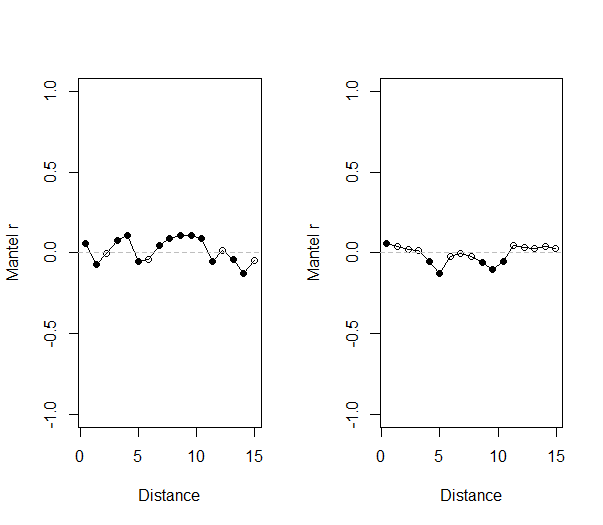


###FIGURE. Build coefplots with estimates with different scale axes for cat and numerical variables

png('./Coefplot.png', width = 4, height = 6, units ='in', res=600)

par(mfcol=c(2,1), mgp=c(2,0.6,0), mai=c(0.7,0,0,0.2), oma=c(0.5,0.5,1.3,0.5), cex.axis=0.9)

coefplot2(LFvsGrper.Biom.full, lwd.1=1, top.axis=T,tcl=0.1,

main=NULL, font.main=1, cex.main=0.9, cex.pts=1.2,

var.idx=c(6,7,8,9,10,11), cex.var=1, mar=c(0,8,2,0), cex.lab=1,

varnames=c('Depth', 'Time since invasion','Reef complexity','Humans/Reef','Predator biomass','Grouper biomass'),

xlab="test", xlim=c(-1,1))

mtext('numerical coeffient estimates', side =3, adj=0.5, cex=1, line=0)

coefplot2(LFvsGrper.Biom.full, lwd.1=1, top.axis=F, tcl=0.1,pch=c(16,15,15,15,15),

main=NULL, cex.main=1, cex.pts=1.2, cex.var=1, cex.axis=0.9,

var.idx=c(1,2,3,4,5), mar=c(3,8,1,0), cex.lab=1,

varnames= c('Intercept','Spur & Groove','Slope','Winward','Protected'),

xlab="categorical coefficient estimates", xlim=c(-7,7))

dev.off()

#####Build Fig showing lionfish vs grouper biomass with prediction

#Calculate prediction for LF abundance vs Grouper biomass when all the cofactors are in.

fish <- na.omit(fish) #omit NAs

newdat <- data.frame(

LF.Count = LF.Count,

Grouper.Biom = fish$Grouper.Biom,

Predators = 0,

Protection = 0,

Habitat = 0,

WindvsLee = 0,

Depth= 0,

Time= 0,

Rugosity.t. = 0,

Hum.Reef = 0,

LogArea=fish$LogArea)

#Calculate prediction of the relationshio between lionfish and grouper when all the covariates have 0 effect.

Prediction <- predict(LFvsGrper.Biom.full, newdata=fish, type="response", interval="confidence")

#But this predicts Counts need to calculate Abundance so divide by area of survey

Predictionfit=Prediction$fit/Area4LF

Predictionlwr=Prediction$lwr/Area4LF

Predictionupr=Prediction$upr/Area4LF

fish$Predictionfit<-Predictionfit; fish$Predictionlwr<-Predictionlwr; fish$Predictionupr<-Predictionupr

#Caculate means for LF biomass ang Grouper biomass

#Add everything as dataframe

#First recode protection, requires car package

library(car)

fish$Protection <- as.numeric(recode(fish$Protection, "'n'='1';'y'='2'"))

newdata<-data.frame(

Grouper.Biom.mean = tapply(fish$Grouper.Biom, fish$Site.Code, mean),

LF.Abund.mean = tapply(fish$LF.Abund,fish$Site.Code, mean),

LF.Biom.mean = tapply(fish$LF.Biom,fish$Site.Code, mean),

Protection.mean = tapply(fish$Protection,fish$Site.Code,mean),

Predictionfit.mean = tapply(fish$Predictionfit,fish$Site.Code, mean),

plwr.mean = tapply(fish$Predictionlwr,fish$Site.Code,mean),

pupr.mean = tapply(fish$Predictionupr,fish$Site.Code,mean))

newdata = na.omit(newdata)

#BUILD figure and save it as png figure

png("./FigLFvsGrp.png",

width = 4, height = 4, units ="in", res =600)

#Load ggplot Library to make pretty graphis

library (ggplot2)

library (gridExtra) # to add panels

# Build GGplot of Grouper ~ LF.Abund and protection status

p1 <- ggplot(newdata, aes (Grouper.Biom.mean,LF.Abund.mean, colour=factor(Protection.mean))) +

xlab(expression('Groupers'~(x10^{2}~g~100~m^{-2}))) +

ylab(expression('Lionfish'~~(x10^{2}~ind~100~m^{-2}))) +

geom_point(pch=16, cex=2.8)+ theme_bw()+ theme(legend.position=c(0.75,0.85),

legend.title=element_blank())+

scale_colour_discrete(name = "",breaks=c("1", "2"),labels=c("Non-protected", "Protected"))+

#legend.text = element_text("Non-Protected", "Protected"))+

theme(panel.grid.minor=element_blank(), panel.grid.major=element_line(colour="grey99"),

axis.title.x=element_text(size=11),axis.title.y=element_text(size=11))+

#Add prediction based on CI on FE uncertainty and RE variance

geom_smooth(aes(ymin=plwr.mean, ymax=pupr.mean),method="glm", formula= y ~log(x+1), col="black")

#Plot graphic wihtout Eleuthera data

newdatanoEleu = newdata[26:71,]

p2 <- ggplot(newdatanoEleu, aes (Grouper.Biom.mean,LF.Abund.mean, colour=factor(Protection.mean))) +

xlab(expression('Groupers'~(x10^{2}~g~100~m^{-2}))) +

ylab(expression('Lionfish'~~(x10^{2}~ind~100~m^{-2}))) +

geom_point(pch=16, cex=2.8)+ theme_bw()+ theme(legend.position=c(0.75,0.85),

legend.title=element_blank())+

scale_colour_discrete(name = "",breaks=c("1", "2"),labels=c("Non-protected", "Protected"))+

#legend.text = element_text("Non-Protected", "Protected"))+

theme(panel.grid.minor=element_blank(), panel.grid.major=element_line(colour="grey99"),

axis.title.x=element_text(size=11),axis.title.y=element_text(size=11))

# close device

dev.off()

p1;p2


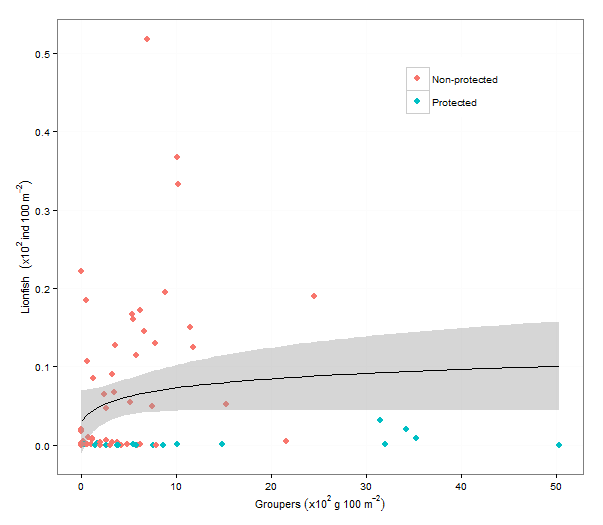


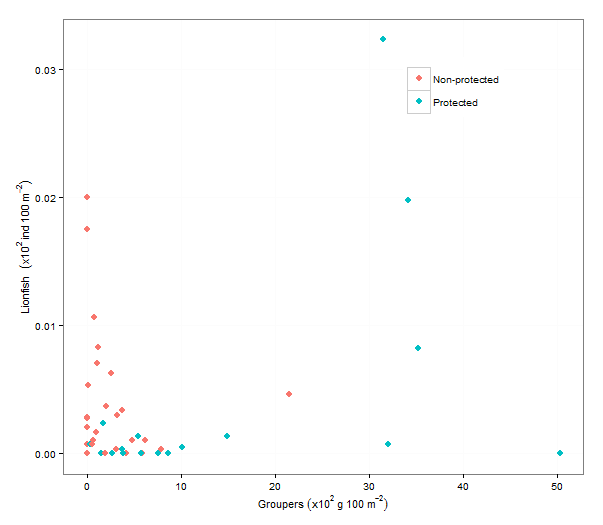


####MAIN FIGURE. LF.Biom ~ large Groupers

####Add data from Mumby et al 2011 as overlaid points

# save it as png format

png("./FigHybridxylog.png",

width =4.27, height = 4, units ="in", res =600)

#Add Mumby's points from Mumby et al 2011 from Plot Digitizer

mgrouper = c(1366.5563, 306.07648, 881.74725, 1418.4756, 2260.742, 3032.3643, 1310.4152, 499.32184, 322.17896, 252.83315, 236.8612, 157.16516)

mLionfish = c(9, 21.68013, 23.990307, 28.432957, 24.168013, 28.610662, 36.60743, 43.36026, 73.39257, 73.74798, 76.946686, 75.880455)

#Build a layout

nf <- layout(matrix(c(2,0,1,3),2,2,byrow = TRUE), c(4,1), c(1,4), TRUE)

layout.show(nf)

#Add scatter plot to layout

par(mar = c(3,3,1,1), mar=c(5,5,0,0))

plot(tapply((fish$LF.Biom+0.09)*100,fish$Site.Code, mean)~tapply((Grouper.Biom+0.09)*100, fish$Site.Code, mean),

col="black",

data=fish,pch=16, log='xy',

xlab = expression("Mean grouper biomass"~(g~100~m^{-2})),

ylab = expression("Mean lionfish biomass"~(g~100~m^{-2})))

#add Mumbys points and regression line

points(mgrouper,mLionfish, col=2, pch=15)

segments(100,80,3200,15, col=2)

#Divide points in Protection vs non-protection

fish$Protection <- as.numeric(recode(fish$Protection, "'n'='0';'y'='1'"))

prot = subset(fish, Protection == 1)

points(tapply((prot$LF.Biom+0.09)*100,prot$Site.Code, mean)~tapply((prot$Grouper.Biom+0.09)*100, prot$Site.Code, mean), col = "grey50", pch=16)

#Add boxplot for grouper and lionfish and

par(mar = c(3,3,1,1), mar=c(0,5,0,0))

boxplot((tapply((Grouper.Biom+0.09)*100, fish$Site.Code, mean)), mgrouper, log="x",

horizontal=TRUE, border=c("black","red"),

pars=list(boxwex=0.3, outwex = 1), axes=FALSE, frame.plot=F, at=c(1,1.4))

points(c(mean(Grouper.Biom)*100,mean(mgrouper)),c(1,1.4), col=c("black","red"), pch=c(16,15))

par(mar = c(3,0,1,1), mar=c(5,0,0,0))

boxplot((tapply((fish$LF.Biom+0.09)*100,fish$Site.Code, mean)), mLionfish,

log="y", border=c("black","red"), pars=list(boxwex=0.3), axes=FALSE,

frame.plot=F, at =c(1,1.4))

points(c(1,1.4),c(mean(fish$LF.Biom)*100, mean(mLionfish)), col=c("black","red"), pch=c(16,15))

# close png device

dev.off()

par(def.par) # reset to default

#Calculate some basic statistics between our data and Mumby et al 2011

#Mean and standard error for grouper

#Build standard error function

se <- function (x) {sd(x)/sqrt(length(x))}

#Calcualte means and standard errors for our data and Mumby et al 2011

mean(Grouper.Biom); se(Grouper.Biom)

[1] 7.557367

[1] 0.8129854

mean(mgrouper/100); se(mgrouper/100) # divide Mumby data by 100 to get gm-2

[1] 10.03728

[1] 2.637477

mean(LF.Biom); se(LF.Biom)

[1] 7.806015

[1] 0.8397299

#mean wihtout Eleuthera region (patch reefs)

mean(LF.Biom[Region!="Eleuthera"]); se(LF.Biom[Region!="Eleuthera"])

[1] 0.7265443

[1] 0.1485036

#mean of Eleuthera region (patch reefs)

mean(LF.Biom[Region=="Eleuthera"]); se(LF.Biom[Region=="Eleuthera"])

[1] 27.49579

[1] 2.107174

mean(mLionfish/100); se(mLionfish/100)

[1] 0.4298479

[1] 0.07224454

mean(LF.Biom[Habitat=="Patch"]);se(LF.Biom[Habitat=="Patch"])

[1] 27.49579

[1] 2.107174

#Test for statistical differences

#Run a Wilcoxon test as a rank sum test with continuity correction

wilcox.test(Grouper.Biom, (mgrouper/100), alternative="less", conf.int=TRUE, correct=TRUE)

Wilcoxon rank sum test with continuity correction

data: Grouper.Biom and (mgrouper/100)

W = 1197, p-value = 0.00231

alternative hypothesis: true location shift is less than 0

95 percent confidence interval:

-Inf -2.36859

sample estimates:

difference in location

-3.060802

#Histograms for Groupers and other predators

#Plot histogram for large groupers and large predators

#for patch reefs and the rest of the forereefs

#First subset data

#Eliminate RE because is shallower but it is not a patch reef

fish1 <- subset (fish, Site.Code!="RE")

#Calculate values per sites (mean) and create data frame

#First recode Depth Category

fish1$DepthCat <- as.numeric(recode(fish1$DepthCat, "'shallow'='0';'deep'='1'"))

#Create data frame with mean values

fish2 <- as.data.frame(cbind(

Grouper.Biom.mean.4hist = tapply(fish1$Grouper.Biom, fish1$Site.Code, mean),

LP.Biom.mean.4hist = tapply(fish1$Predators, fish1$Site.Code, mean),

Grouper.Abund.mean.4hist = tapply(fish1$Grouper.Abund, fish1$Site.Code, mean),

LP.Abund.mean.4hist = tapply(fish1$LP.Abund, fish1$Site.Code, mean),

DepthCat.4hist = tapply(fish1$DepthCat, fish1$Site.Code, mean)))

fish2 <- na.omit(fish2)

#Calculate the means of the groups and SE of the groups

library(plyr)

groupmean <- ddply(fish2, "DepthCat.4hist", summarise, Biomass.mean=mean(Grouper.Biom.mean.4hist))

groupSE <- ddply(fish2, "DepthCat.4hist", summarise, Biomass.mean=sd(Grouper.Biom.mean.4hist)/sqrt(70))

LPmean <- ddply(fish2, "DepthCat.4hist", summarise, LP.mean=mean(LP.Biom.mean.4hist))

LPmeanSE <- ddply(fish2, "DepthCat.4hist", summarise, LP.mean=mean(LP.Biom.mean.4hist)/sqrt(70))

summarydata <- ddply(fish, "Site.Code", summarise,

Grouper=mean(Grouper.Biom), Lionfish Bioma=mean(LF.Biom))

#Build png figure...

library(ggplot2); library(gridExtra)

png("./DensityPlot.png", width = 6, height = 2.8, units ="in", res =600)

par(mfrow=c(2,2))

h1 <- ggplot (fish2, aes(x=Grouper.Biom.mean.4hist, fill=as.factor(DepthCat.4hist)))+

geom_density(alpha=.4) + ylim(c(0,0.10))+

xlab(expression('Large groupers'~(x10^{2}~g~100~m^{-2}))) + ylab('Density')+

#geom_histogram (binwidth=2, alpha=0.5, position="dodge")+

theme_bw()+ theme(panel.grid.minor=element_blank(), panel.grid.major=element_line(colour="grey97"),

axis.title.x=element_text(size=10),axis.title.y=element_text(size=11))+

theme(legend.position="none", legend.title=element_blank())+

scale_fill_discrete(name = "", breaks=c("1", "2"), labels=c("patch reef", "fore reef"))+

#add group mean

geom_vline(data=groupmean, aes(xintercept=Biomass.mean, colour=as.factor(DepthCat.4hist)), linetype="dashed", size=1)

h2 <- ggplot (fish2, aes(x=LP.Biom.mean.4hist, fill=as.factor(DepthCat.4hist)))+

geom_density(alpha=.4) + ylim(c(0,0.05))+

xlab(expression('Large predators'~(x10^{2}~g~100~m^{-2}))) + ylab('')+

#geom_histogram (binwidth=2, alpha=0.5, position="dodge")+

theme_bw()+ theme(panel.grid.minor=element_blank(), panel.grid.major=element_line(colour="grey97"),

axis.title.x=element_text(size=10),axis.title.y=element_text(size=11))+

theme(legend.position=c(0.75,0.8), legend.title=element_blank())+

scale_fill_discrete(name = "", breaks=c("1", "2"), labels=c("patch reef", "fore reef"))+

#add group mean

geom_vline(data=LPmean, aes(xintercept=LP.mean, colour=as.factor(DepthCat.4hist)), linetype="dashed", size=1)

grid.arrange(h1, h2, widths = c(1,1), ncol=2,nrow=1); dev.off()

#Build histograms for grouper class sizes, for total, protection and among region

grouper <- read.csv("./GrouperSize.csv")

grouper <- na.omit(grouper)

grouper <- subset(grouper, Year!=2008); grouper <- subset(grouper, Year!=2013)

#Calculate total frequencies

a= c(8:23)

values=0

for (i in a) {

values <- c(values, sum(grouper[,i]))

}

total = sum(grouper[,8:23])

Freq=values/total*100

#Calculate frequencies between protection levels

grouperHi <- subset (grouper, Site.Code== "CF" | Site.Code=="PP" | Site.Code=="EP"| Site.Code=="HM"| Site.Code=="AN" | Site.Code== "93" | Site.Code== "RP" | Site.Code== "77" | Site.Code== "MC" | Site.Code== "80"| Site.Code== "55" | Site.Code== "108" | Site.Code== "91"| Site.Code== "BCS")

grouperLo <- subset (grouper, Site.Code!= "CF" | Site.Code!="PP" | Site.Code!="EP"| Site.Code!="HM"| Site.Code!="AN" | Site.Code!= "93" | Site.Code!= "RP" | Site.Code!= "77" | Site.Code!= "MC" | Site.Code!= "80"| Site.Code!= "55" | Site.Code!= "108" | Site.Code!= "91"| Site.Code!= "BCS")

grouperN <- subset(grouper, Protection.Code=="N")

grouperR <- subset(grouper, Protection.Code=="R")

classSize <- c("00-05","06-10","11-20","21-30","31-40","41-50","51-60", "61-70",

"71-80","81-90","91-100","101-110","111-120","121-130",

"131-140","141-150")

TL <- c(2.5,8,15,25,35,45,55,65,75,85,95,105,115,125,135,145)

#Calculate frequencies among habitats

grouperSG <- subset (grouper, Habitat=="Spur and Grove")

grouperSlope <- subset (grouper, Habitat=="Slope" | Habitat=="Border Drop")

grouperPatch <- subset (grouper, Habitat=="Patch Reef")

# For non-protected sites

vectorN=0

for (i in a) {

vectorN <- c(vectorN, sum(grouperN[,i]))

}

total = sum(grouperN[,8:23])

FreqN=vectorN/total*100

# For protected sites

vectorR=0

for (i in a) {

vectorR <- c(vectorR , sum(grouperR[,i]))

}

total = sum(grouperR[,8:23])

FreqR=vectorR/total*100

# For sites with high grouper biomass (min 10 g m2)

vectorHi=0

for (i in a) {

vectorHi <- c(vectorHi, sum(grouperHi[,i]))

}

total = sum(grouperHi[,8:23])

FreqHi=vectorHi/total*100

FreqHi=FreqHi[-1]

# For sites with lower grouper biomass (min 10 g m2)

vectorLo=0

for (i in a) {

vectorLo <- c(vectorLo, sum(grouperLo[,i]))

}

total = sum(grouperLo[,8:23])

FreqLo=vectorLo/total*100

FreqLo=FreqLo[-1]

# For Patch

Patch=0

for (i in a) {

Patch <- c(Patch, sum(grouperPatch[,i]))

}

total = sum(grouperPatch[,8:23])

FreqPatch=Patch/total*100

FreqPatch=FreqPatch[-1]

# For Spur and Groove

SG=0

for (i in a) {

SG <- c(SG, sum(grouperSG[,i]))

}

total = sum(grouperSG[,8:23])

FreqSG=SG/total*100

FreqSG=FreqSG[-1]

# For Slope

Slope=0

for (i in a) {

Slope <- c(Slope, sum(grouperSlope[,i]))

}

total = sum(grouperSlope[,8:23])

FreqSlope=Slope/total*100

FreqSlope=FreqSlope[-1]

# Create matrix

Size <- (cbind(FreqN=FreqN[-1],FreqR=FreqR[-1]))

SizeLH <- (cbind(FreqLo,FreqHi))

SizeHab <- cbind(FreqSG,FreqSlope,FreqPatch)

#transpose matrix Size

Sizet=as.matrix(t(Size))

SizeLHt=as.matrix(t(SizeLH))

SizeHab=as.matrix(t(SizeHab))

#Assign column names

colnames(Sizet) <- classSize

colnames(SizeLHt) <- classSize

colnames(SizeHab) <- classSize

#calculate grouper size average and standard error per protection status

#Mean and SE for non-protectec sites

SizeN <- sum(TL*Size[,1]/sum(Size[,1]))

SizeNsd <- sqrt(sum(Size[,1]*(TL-SizeN)^2)/(sum(Size[,1])-1))

SizeNse <- sqrt(sum(Size[,1]*(TL-SizeN)^2)/(sum(Size[,1])-1))/sqrt(sum(Size[,1]))

#Mean and SE for Protected sites

SizeP <- sum(TL*Size[,2])/sum(Size[,2])

SizePsd <- sqrt(sum(Size[,2]*(TL-SizeP)^2)/(sum(Size[,2])-1))

SizePse <- sqrt(sum(Size[,2]*(TL-SizeP)^2)/(sum(Size[,2])-1))/sqrt(sum(Size[,2]))

#Check mean and SE values

SizeN #mean grouper non protected sites

[1] 34.70988

SizeNse #se for grouper non protected sites

[1] 1.045044

SizeP # mean grouper protected sites

[1] 48.55932

SizePse #se for grouper protected sites

[1] 1.468877

#Compare means, but build function first

ttest <- function (mu,s,n) { -diff(mu)/sqrt(sum(s^2/n))}

mu = c(SizeN,SizeP); s = c(SizeNsd,SizePsd); n=c(sum(Size[,1]),sum(Size[,2]))

t<- ttest(mu,s,n); t

[1] -7.682625

#calculate p values for ttest

pt(t, df=pmin(sum(Size[,1]),sum(Size[,2])-1))

[1] 5.704656e-12

#Bar plots

png("./Hist3.png", width = 6, height = 6, units ="in", res =600)

par(mfrow=c(3,1), mai=c(0.1,0.8,0.1,0.1), mgp=c(2,0.7,0),

oma=c(4,0.8,0.5,0), cex=1)

barplot(Sizet,beside = T, width = 3, ylim=c(0,40), cex.axis=0.9,cex.names=0.8, space=c(0,0.2),

ylab="frequency %",axisname=F, tcl=-0.3,

legend.text=c("Non-protected", "Protected"), bty="n", las=1)

mtext("A", side=3, adj=-0.12, line =-0.5, las=1)

barplot(SizeLHt,beside = T, width = 3, ylim=c(0,40),

cex.axis=0.9,cex.names=0.8, space=c(0,0.2),axisname=F,

ylab="frequency %", tcl=-0.3,

legend.text=c("Sites with groupers <10g/m^2", "Sites with groupers >10g/m^2"), las=1, bty="n")

mtext("B", side=3, adj=-0.12, line =-0.5, las=1)

barplot(SizeHab,beside = T, width = 3, ylim=c(0,50),

cex.axis=0.9, cex.names=0.9, space=c(0,0.2), col=c("grey25", "grey75", "white"),

ylab="frequency %", tcl=-0.3,

legend.text=c("Spur & Groove", "Slope", "Patch Reef"), las=1)

mtext("class size (cm)", side=1, adj=0.5, line=2.5)

mtext("C", side=3, adj=-0.12, line=-0.5, las=1)

dev.off()
